# Transcriptomic profiles of aging in purified human immune cells

**DOI:** 10.1186/s12864-015-1522-4

**Published:** 2015-04-22

**Authors:** Lindsay M Reynolds, Jingzhong Ding, Jackson R Taylor, Kurt Lohman, Nicola Soranzo, Alberto de la Fuente, Tie Fu Liu, Craig Johnson, R Graham Barr, Thomas C Register, Kathleen M Donohue, Monica V Talor, Daniela Cihakova, Charles Gu, Jasmin Divers, David Siscovick, Gregory Burke, Wendy Post, Steven Shea, David R Jacobs, Ina Hoeschele, Charles E McCall, Stephen B Kritchevsky, David Herrington, Russell P Tracy, Yongmei Liu

**Affiliations:** Department of Epidemiology and Prevention, Division of Public Health Sciences, Wake Forest School of Medicine, Winston-Salem, North Carolina 27157 USA; Department of Internal Medicine, Wake Forest School of Medicine, Winston-Salem, North Carolina 27157 USA; Department of Gerontology and Geriatric Medicine, J. Paul Sticht Center on Aging, Wake Forest School of Medicine, Winston-Salem, North Carolina 27157 USA; Department of Biostatistical Sciences, Division of Public Health Sciences, Wake Forest School of Medicine, Winston-Salem, North Carolina 27157 USA; CRS4 Bioinformatica, Pula, 09010 Italy; FBN, Leibniz Institute for Farm Animal Biology, Genetics and Biometry, Mecklenburg-Vorpommern, Germany; Departments of Medicine and Epidemiology, Cardiovascular Health Research Unit, University of Washington, Seattle, Washington 98115 USA; Departments of Medicine and Epidemiology, Columbia University, New York, New York 10032 USA; Department of Pathology, Wake Forest School of Medicine, Winston-Salem, North Carolina 27157 USA; Department of Pathology, Johns Hopkins University, Baltimore, Maryland 21205 USA; Division of Biostatistics, Washington University School of Medicine, St. Louis, Missouri 63110 USA; New York Academy of Medicine, New York, New York 10029 USA; Division of Epidemiology and Community Health, School of Public Health, University of Minnesota, Minneapolis, Minnesota 55454 USA; Virginia Bioinformatics Institute, Virginia Tech, Blacksburg, Virginia 24061 USA; Department of Molecular Medicine, Wake Forest School of Medicine, Winston-Salem, North Carolina 27157 USA; Department of Pathology, University of Vermont, Colchester, Vermont 05446 USA

**Keywords:** Aging, Monocyte, T cell, Transcriptome, Mitochondrial ribosome, Translation, Protein synthesis, Ribonucleoprotein complex, Oxidative phosphorylation, Autophagy, Methylation

## Abstract

**Background:**

Transcriptomic studies hold great potential towards understanding the human aging process. Previous transcriptomic studies have identified many genes with age-associated expression levels; however, small samples sizes and mixed cell types often make these results difficult to interpret.

**Results:**

Using transcriptomic profiles in CD14+ monocytes from 1,264 participants of the Multi-Ethnic Study of Atherosclerosis (aged 55–94 years), we identified 2,704 genes differentially expressed with chronological age (false discovery rate, FDR ≤ 0.001). We further identified six networks of co-expressed genes that included prominent genes from three pathways: protein synthesis (particularly mitochondrial ribosomal genes), oxidative phosphorylation, and autophagy, with expression patterns suggesting these pathways decline with age. Expression of several chromatin remodeler and transcriptional modifier genes strongly correlated with expression of oxidative phosphorylation and ribosomal protein synthesis genes. 17% of genes with age-associated expression harbored CpG sites whose degree of methylation significantly mediated the relationship between age and gene expression (p < 0.05). Lastly, 15 genes with age-associated expression were also associated (FDR ≤ 0.01) with pulse pressure independent of chronological age.

Comparing transcriptomic profiles of CD14+ monocytes to CD4+ T cells from a subset (n = 423) of the population, we identified 30 age-associated (FDR < 0.01) genes in common, while larger sets of differentially expressed genes were unique to either T cells (188 genes) or monocytes (383 genes). At the pathway level, a decline in ribosomal protein synthesis machinery gene expression with age was detectable in both cell types.

**Conclusions:**

An overall decline in expression of ribosomal protein synthesis genes with age was detected in CD14+ monocytes and CD4+ T cells, demonstrating that some patterns of aging are likely shared between different cell types. Our findings also support cell-specific effects of age on gene expression, illustrating the importance of using purified cell samples for future transcriptomic studies. Longitudinal work is required to establish the relationship between identified age-associated genes/pathways and aging-related diseases.

**Electronic supplementary material:**

The online version of this article (doi:10.1186/s12864-015-1522-4) contains supplementary material, which is available to authorized users.

## Background

Identifying molecular features that vary with chronological age has critical implications for our understanding of aging and the development of age-associated diseases. A number of previous studies have performed systematic investigations of the relationship between age and gene expression in various human tissues, including T cells [1-3], whole blood [4], peripheral blood mononuclear cells (PBMCs) [5], brain [6-8], and muscle tissue [7,9]. Although very few individual genes with age-associated expression have been identified across studies or tissues [8], similar gene functions/pathways have been reported. For instance, pathway analyses of age-associated genes identified an enrichment of immune function and inflammation genes in various mixtures of blood cells [2-5]. Other aging-associated pathways/processes found to be enriched in blood as well as brain and muscle tissues include RNA processing [6-8,10] and chromatin remodeling [6,7,10], while mitochondrial pathways [6,8] and more specifically the oxidative phosphorylation/electron transport pathway [7,9] were detectable in studies of skin, brain, and muscle tissues. However, interpretation of these findings is limited by small sample sizes and often heterogeneous cellular composition of the samples investigated. Currently, there is a lack of well-powered transcriptomic studies of aging using homogeneous cell samples.

CD4+ T cells and CD14+ monocytes are excellent cell types for transcriptomic studies of aging in humans. T cells and monocytes can be isolated from an easily accessible tissue (blood), and both have known roles in the development of age-related diseases. T cells are well known to exhibit numerous functional impairments with advanced age and have been implicated in the age-dependent decline in immune function, commonly known as immunosenescence [11]. To date, the largest aging transcriptomic study of CD4+ T cells included 31 individuals, aged 25 – 81 years, from the Baltimore Longitudinal Study of Aging. Comparison of T cell expression profiles from individuals less than 65 years of age to those age 65 and older revealed 264 genes associated with age (p < 0.05, FDR < 0.3) [3]. Monocytes have also been shown to exhibit phenotypic and functional changes in the elderly [12], and are key cells of innate immunity and major contributors to the pathogenesis of inflammatory and degenerative diseases [13]. To our knowledge the effect of aging on the monocyte transcriptome has not yet been investigated.

Previously, we purified CD14+ monocytes from the peripheral blood of 1,264 participants of the Multi-Ethnic Study of Atherosclerosis (MESA). We measured both genome-wide gene expression and DNA methylation in these purified monocyte samples using microarrays, and identified cytosine-guanine dinucleotides (CpGs) whose degree of methylation was associated with *cis*-gene expression (FDR < 0.001) [14]. Given that DNA methylation can vary with age [15-20], we also investigated the relationship between age and DNA methylation in the 1,264 MESA monocyte samples [21], and identified 1,794 CpGs whose degree of methylation was associated with age and *cis*-gene expression (FDR < 0.001) [21]. However, it remains unknown to what extent age-related variations in the methylome may mediate the overall effect of age on gene expression.

In response to these uncertainties, here we present a comprehensive analysis of age and gene expression in the CD14+ monocyte samples from 1,264 MESA participants ranging in age from 55 to 94 years. Additionally, we present an analysis of age and gene expression in circulating CD4+ T cells from a subset of 423 MESA participants. This cohort study offers the unique opportunity to 1) better understand the effect of aging on gene expression in CD14+ monocytes and CD4+ T cells, 2) compare the aging transcriptome measured in two cell types from the same individuals, 3) investigate CpG methylation as a potential mediator of age-associated variations in the transcriptome, and 4) characterize the relationships between chronological age-associated gene expression and a clinical measure of vascular age, pulse pressure.

## Results and discussion

**Figure 1:**
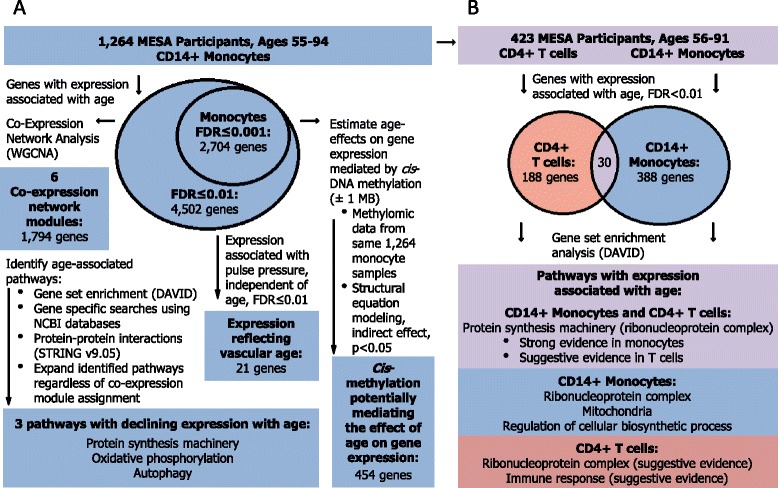
Study design and results summary. **A)** CD14+ monocytes were purified from 1,264 peripheral blood samples by magnetic bead selection, and gene expression levels were measured using microarray. Age-associated expression (FDR ≤ 0.01) was detected for 4,502 genes, which were further analyzed using the indicated *in silico* approaches to identify and investigate potential age-related pathways. Results support a potential transcriptomic decline in ribosomal protein synthesis machinery, as well as declines in oxidative phosphorylation and autophagy gene expression with age. Measures of DNA methylation and pulse pressure were incorporated to investigate DNA methylation as a potential mediator for the effect of age on gene expression, and to prioritize age-associated gene expression for potential relevance to vascular age. **B)** CD4+ T cells were purified in a subset of the peripheral blood samples by magnetic bead selection, and gene expression levels were measured using microarray. Age-associated genes (FDR < 0.01) were identified, revealing 30 genes with expression significantly associated with age in both monocytes and T cells from the same individuals. No pathways were significantly (FDR < 0.05) enriched among age-associated genes in T cells; however, suggestive evidence was observed for the ribonucleoprotein complex and immune response pathways.

The overall study design and results are summarized in Figure 1, and the population characteristics are described in Additional file 1: Table S1.

### Transcriptomic profiles associated with age in 1,264 monocytes samples

Transcriptomic profiling of CD14+ monocytes using microarrays (Illumina HumanHT-12 v4 Expression BeadChip) revealed 10,898 expressed genes, of which 25% had expression significantly (FDR ≤ 0.001, p < 9.0×10^−4^) associated with chronological age (Figure 1A, and Additional file 1: Figure S1) after adjusting for appropriate biological and technical covariates including race, gender, study site, and estimates of residual sample contamination with non-targeted cell types (see Methods). The effect size of a ten-year difference in age for individual gene expression was modest (up to 10%). The majority of the associations with age remained significant in the “disease free” (no report of diabetes, cancer, or cardiovascular diseases; n = 839), sex- and ethnic-specific subgroups (Additional file 2: Table S2).

Gene set enrichment analysis [22,23] identified the ribonucleoprotein complex, mitochondrial ribosome, and oxidative phosphorylation pathway genes enriched among age-associated genes. After stratifying by the effect direction of age on expression, the genes with expression negatively associated with age were found to be enriched with ribosomal/translation and mitochondrial/oxidative phosphorylation genes (Additional file 1: Table S3). In contrast, genes with expression positively associated with age were enriched with pathways relating to regulation of transcription, the cytoskeleton, protein phosphorylation, and response to insulin.

### Co-expression network analysis

**Figure 2:**
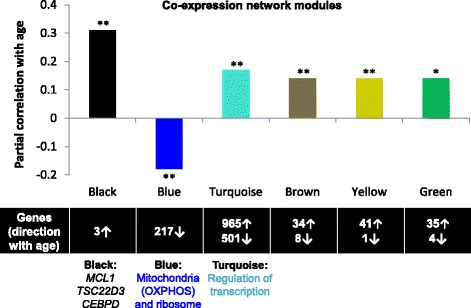
Co-expression network modules associated with chronological age. Six mutually exclusive gene network modules with coordinate gene expression profiles associated with chronological age were identified in 1,264 monocyte samples (using WGCNA), ranging in size from 3 to 1,466 genes. For each module (x-axis), the partial correlation between age and the module eigengene is shown (y-axis); covariates included race, sex, site of data collection, and residual sample contamination with non-targeted cells. Below each module is the number of genes assigned to the module, and the direction of expression association with age; network modules discussed in further detail include the ‘black’ module (containing three genes: *MCL1, TSC22D3,* and *CEBPD*), and the ‘blue’ and ‘turquoise’ modules (which were significantly enriched with age-related pathways shown in Table 1). The significance of the module eigengene association with age is denoted as: * p < 0.008 (Bonferroni adjusted significance threshold for testing six modules, alpha = 0.05), and ** p ≤ 1x10^−6^.

To identify consensus networks of genes with coordinated expression profiles associated with age, we used a weighted gene co-expression network analysis [24] (WGCNA), combined with a stability analysis (see Methods), and examined an expanded set of 4,502 genes associated with age at a more liberal FDR threshold of ≤ 0.01 (Figure 1A and Additional file 2: Table S2). Six mutually exclusive gene network modules were identified, labeled as colors: ‘black’, ‘blue’, ‘turquoise’, ‘brown’, ‘yellow’, and ‘green’. Network modules ranged in size from three to 1,466 genes, and had significant module eigengene (1st eigenvector) associations with age with p ranging from 1.8×10^−30^ to 1.3×10^−6^ (Figure 2 and Additional file 1: Table S4). Genes assigned to the same module had moderately to strongly correlated expression (absolute median r ranging 0.41 – 0.62). To better understand the relationships between modules, we examined the correlations between eigengenes of each module, and found a very strong negative correlation between the ‘blue’ and ‘turquoise’ module eigengenes (correlation = −0.90; Additional file 1: Figure S2).

Gene set enrichment analysis revealed significantly (FDR < 0.05) enriched pathways within two of the six modules, relative to all monocyte expressed genes (Table 1). The 217 genes assigned to the ‘blue’ module, all with expression negatively associated with age, were enriched with 1) ribonucleoprotein complex genes (including translation, ribosome biogenesis, and RNA processing genes) and 2) mitochondrion genes (including oxidative phosphorylation and mitochondrial ribosome genes). The largest gene network module, the ‘turquoise’ module, was assigned 1,466 genes which were significantly enriched with nuclear lumen genes, many of which with known roles in RNA splicing and the regulation of transcription.

**Table 1:** **Significantly enriched Gene Ontology terms among co-expression network modules in CD14+ monocytes**

**Gene Ontology term**	**Gene Count**	**Direction of age effect**	**Fold Enrichment**	**Nominal P-value**	**FDR**
**‘Blue’ network module** ***(*** **217 genes; 217↓0↑)**					
*Ribonucleoprotein complex:*	58	(58↓0↑)	5.6	5.71E-29	7.40E-26
Translation	41	(41↓0↑)	6.9	3.48E-23	5.20E-20
Ribosome biogenesis	16	(16↓0↑)	7.1	4.57E-09	6.84E-06
RNA processing	26	(26↓0↑)	2.8	5.25E-06	7.86E-03
*Mitochondrion:*	61	(61↓0↑)	3.2	5.67E-18	7.36E-15
Oxidative phosphorylation	15	(15↓0↑)	10.0	1.57E-10	2.35E-07
Respiratory chain complex I	8	(8↓0↑)	11.3	4.55E-06	5.90E-03
*‘* **Turquoise’ network module (1,466 genes; 501↓965↑)**					
*Nuclear lumen:*	199	(48↓151↑)	1.4	9.05E-07	1.32E-03
RNA splicing	59	(17↓42↑)	1.8	1.43E-05	2.54E-02

Results from gene set enrichment analysis are presented (from DAVID) for co-expression network modules with significantly enriched Gene Ontology (GO) terms (FDR < 0.05). No significant enrichment was observed among the ‘black’, ‘brown’, ‘yellow’, or ‘green’ network module genes; background genes included all 10,898 genes with expression detectable in 1,264 CD14+ monocyte samples.

↑ genes with expression positively associated with age; ↓ genes with expression negatively associated with age.

The other four co-expression network modules (shown in Figure 2 as ‘black’, ‘brown’, ‘yellow’, and ‘green’) were found to have weakly to moderately correlated eigengenes (Additional file 1: Figure S2) which were positively associated with older age. No significantly enriched pathways were detected within these four modules with a false discovery rate of 5%; however, there were pathways enriched with nominal significance among these modules, including insulin signaling (‘brown’, fold enrichment = 19.9, p = 8.88×10^−4^), immune response (‘yellow’, fold enrichment = 5.1, p = 1.97×10^−4^), and regulation of apoptosis (‘green’, fold enrichment 3.9, p = 1.12×10^−3^).

### Autophagy-related gene expression

Autophagy is a degradation pathway that utilizes double-membrane vesicles, termed autophagosomes, to engulf long-lived proteins, damaged organelles, and invasive pathogens, and to transport these cargos to the lysosomes for degradation [25]. In the aging field, impaired autophagy is considered one of the principal determinants of cellular aging, which is supported by *in vitro* and animal study findings that autophagy declines with age [26]. However, studies of autophagy and age in humans are sparse.

One of the most significant age-gene expression associations we observed in monocytes from 1,264 individuals was with *MCL1 (*myeloid cell leukemia sequence 1; FDR = 7.6×10^−16^). *MCL1,* a known inhibitor of autophagy and apoptosis, is a member of the Bcl-2 (B-cell CLL/lymphoma 2) family, which includes many other proteins known to regulate autophagy and apoptosis [27-29]. The positive relationship between *MCL1* expression and age tends to be linear across the range of ages (55 – 94 years) in this population (Additional file 1: Figure S3). We confirmed an age-associated increase in *MCL1* mRNA expression in a subset of the population using RNA sequencing technology (n = 373; p = 2.98×10^−5^; Additional file 1: Figure S4). *MCL1* gene expression was also significantly correlated with MCL1 protein expression measured in a subset of the population using Western Blot for (n = 30, r = 0.42; p-value = 0.02; Additional file 1: Figure S5).

*MCL1* was assigned to the co-expression network module whose eigengene was most significantly associated with age (‘black’, p_eigengene_ = 1.79×10^−30^). In addition to *MCL1*, the ‘black’ module contained two other genes with expression positively associated with age – *TSC22D3* (TSC22 domain family, member 3; FDR = 6.69×10^−24^) and *CEBPD* (CCAAT/enhancer binding protein, delta; FDR = 3.82×10^−15^)*,* which encode transcription factors involved in the suppression of inflammation and apoptosis [30,31]. While a common regulator for these three ‘black’ module genes has not been identified, the limited literature available points towards cytokines such as IL-2 (Interleukin 2) and IL-6 in the up-regulation of ‘black’ module gene expression, possibly through the activation of STAT proteins [30,32-34]. Notably, STATs 1, 3, 4, and 5A were also found in our list of genes that increase expression with age (FDR = 3.59 ×10^−6^, 5.40 ×10^−7^, 6.46 ×10^−5^, and 2.49×10^−3^, respectively).

**Figure 3:**
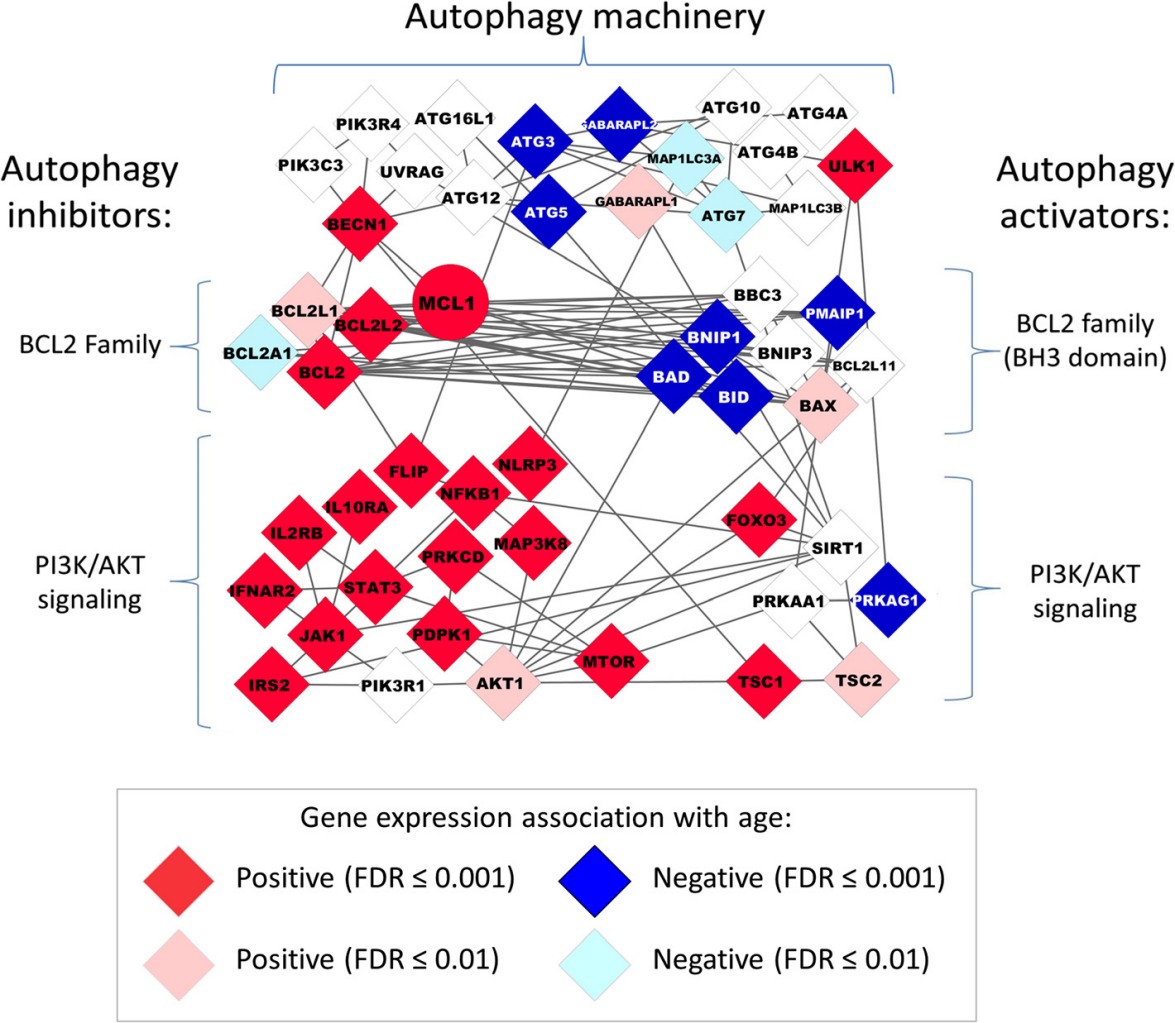
Age-associated expression pattern for the Bcl-2 family and other key autophagy genes suggest autophagy declines with age. The ‘black’ co-expression network module gene - *MCL1* (circle), and other key genes (diamonds) encoding autophagy machinery and autophagy inhibitors/activators (related to the Bcl-2 family and the PI3K/AKT signaling pathway) are shown, with edges representing previously characterized protein-protein interactions (STRING v9.05). Color denotes the direction and significance resulting from the association of age and gene expression in 1,264 CD14+ human monocyte samples, adjusting for race, sex, study site, and residual cell contamination with other cell types.

Given the limitation of the WGCNA network analysis (hierarchical clustering only allows single module membership), and the known role for MCL1 in the inhibition of autophagy [29], we next examined the relationship between age and expression for key autophagy genes disregarding network module membership. The associations of age and gene expression, as well as the previously characterized protein-protein interactions [35], are shown for key autophagy genes in Figure 3. Among the well-known regulators of autophagy within the Bcl-2 family [36], age was positively associated with expression of inhibitors of autophagy (i.e. *MCL1*, *BCL2*, and *BCL2L2;* FDR: 7.60×10^−16^ – 1.15×10^−3^), and negatively associated with expression of activators of autophagy (i.e. *BAD* and *BID;* FDR: 8.28×10^−7^ and 1.18×10^−4^, respectively). Negative effects of age on gene expression were also observed for genes which encode proteins critical for autophagosome formation [26], including autophagy machinery genes *ATG3, ATG5, ATG7*, and *GABARAPL2* (FDR ranging 3.48×10^−4^ – 1.8×10^−3^). Additionally, we observed a positive effect of age on the expression of autophagy inhibitors belonging to the PI3K/Akt signaling pathway (*MTOR, IL10RA, STAT3, JAK1, PDPK1, IL2RB;* FDR ranging 1.45×10^−8^ - 9.88×10^−4^), while negative effects of age were observed for a PI3K/Akt signaling pathway gene important for autophagy activation [37,38], AMPK (*PRKAG1*, FDR = 4.87x10^−7^). However, exceptions to the apparent age-dependent transcriptional decline of autophagy gene expression were also observed, such as increasing expression of pro-autophagy genes [39], *BECN1* (Beclin-1, autophagy related; FDR = 1.33×10^−4^) and *ULK1* (unc-51-like kinase 1; FDR = 9.97×10^−5^) with older age.

The protein networks that regulate autophagy and apoptosis are highly interconnected, and crosstalk has been observed, particularly among Bcl-2 family members [36]. However, an overall transcriptional decline in apoptosis gene expression with age was not apparent, as other key regulators of the apoptotic pathway, such as pro-apoptotic *CASP2*, *CASP8*, and *FOXO3*, had increasing expression associated with older age (FDR = 3.9×10^−4^, 4.5×10^−3^, and 6.0×10^−8^, respectively).

*In vitro* and animal studies have reported a decline in autophagy with age [26,36,40-43]; however, to our knowledge, only one other publication has reported an age-associated decline in expression of autophagy genes, which was carried out in a small number of human brain tissue samples [44]. Overall, these findings for major components of core autophagy machinery and upstream regulators provide evidence for a transcriptional decline in autophagy gene expression with age in human monocytes. The identification of key genes contributing to a decline in autophagy are of great interest, as pharmacologic activation of autophagy has been linked with increasing lifespan in animal models, including mice [45]. Further, dysfunctional autophagy is now widely implicated in pathophysiological processes of many age-related diseases such as cancer, Alzheimer’s, diabetes, and cardiovascular diseases [46]. However, longitudinal studies are necessary to validate the age-related transcriptional decline of autophagy gene expression in human monocytes, and to investigate the relationship between these age-related patterns and the development of age-associated diseases.

### Oxidative phosphorylation and protein synthesis machinery gene expression

**Figure 4:**
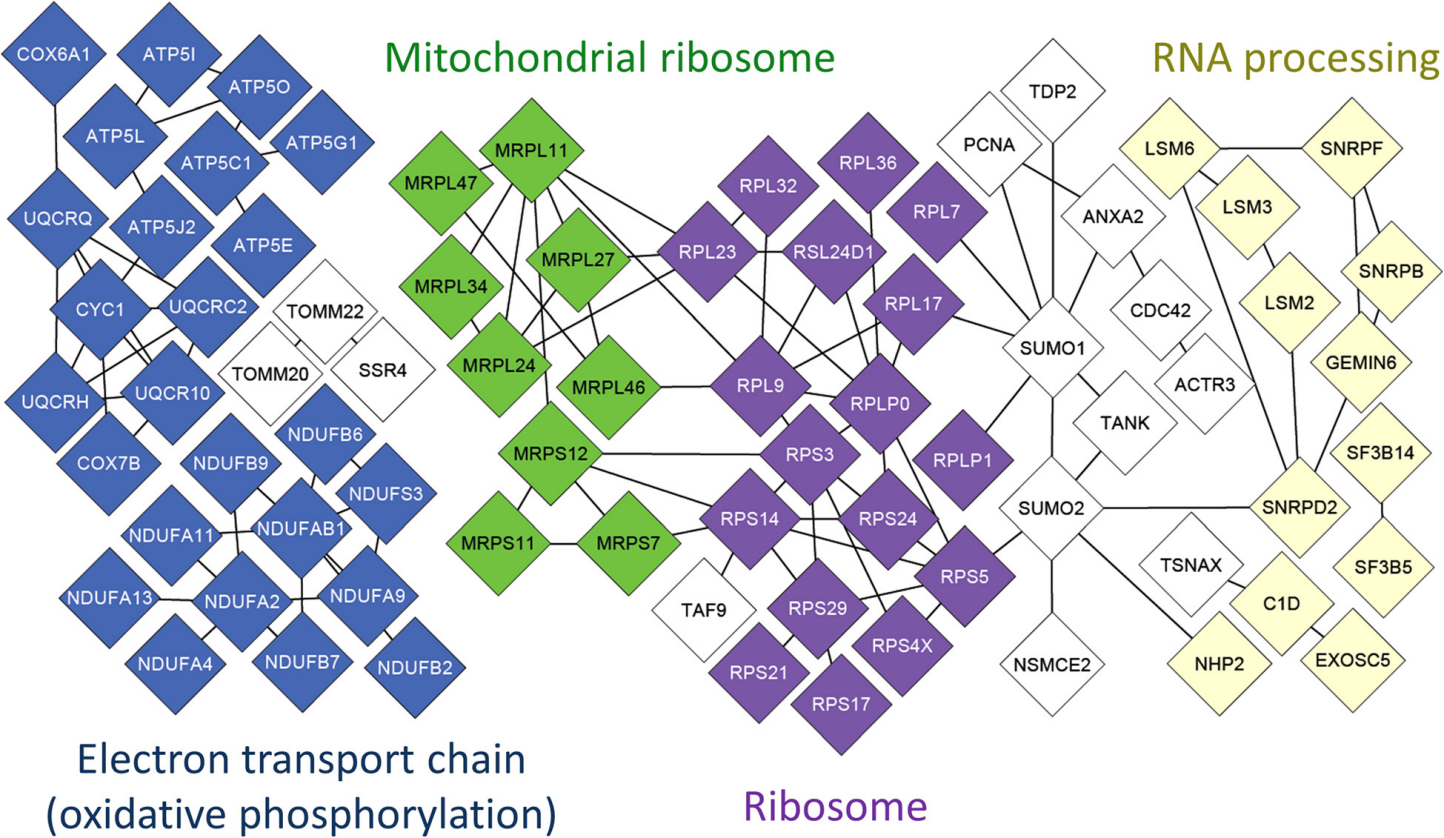
Protein functions and interactions between co-expressed genes assigned to the ‘blue’ network module. In 1,264 monocyte samples, older age was associated with lower expression of 217 co-expressed genes assigned to the ‘blue’ network module, 77 of which (shown as diamonds) have experimental evidence for interaction with other ‘blue’ module genes (interactions shown as edges, from STRING v9.1). Color denotes gene membership to Gene Ontology (GO) pathways enriched in the ‘blue’ module relative to all monocyte expressed genes (FDR < 0.05), including the electron transport chain/oxidative phosphorylation pathway (blue) and ribonucleoprotein complex pathway (green, purple, and yellow) – comprised of protein synthesis machinery genes from the mitochondrial ribosome (green), the ribosome (purple), and RNA processing genes (yellow). Genes relating to other cellular processes (white) include mitochondrial protein import genes (*TOMM20, TOMM22*) and DNA damage response genes (*NSMCE2, SUMO2, SUMO1, TDP2*); ‘blue’ module genes without reported protein-protein interactions are not shown (140 genes).

According to the mitochondrial theory of aging, mitochondria are among the key players contributing to the aging process, whose dysfunction is linked with aging [47] and age-related diseases [48,49]. Consistent with previous findings from multiple human tissues and across species [50], our data revealed a pattern of decreasing expression of mitochondrial oxidative phosphorylation (OXPHOS) genes with age in monocytes, particularly among genes within the ‘blue’ network module (Table 1). The ‘blue’ module genes were also enriched with ribonucleoprotein complex genes. Upon examining ‘blue’ module genes for previously characterized protein-protein interactions (Figure 4), two sub-networks were identified: one relating to the mitochondrial electron transport chain, and the other composed of ribonucleoprotein complex genes. The majority of the ribonucleoprotein complex genes were ribosomal protein synthesis machinery genes.

To better understand the relationship between age and global expression of OXPHOS and ribosomal protein synthesis genes, we examined the associations between age and expression of all OXPHOS (54 genes, GO:0006119), ribosome (204 genes, GO:005840), and mitochondrial ribosomal genes (51 genes, GO:0005761) expressed in monocytes, disregarding network module membership. Overall, we found almost two-thirds of the expressed OXPHOS genes (61%) and ribosomal genes (67%) had expression negatively associated with age (FDR ≤ 0.01, Additional file 2: Table S5 and Additional file 2: Table S6). Among the mitochondrial ribosomal genes, 80% had expression negatively associated with age (FDR ≤ 0.01) (Additional file 2: Table S7). Using western blot to measure the protein levels of one mitochondrial ribosomal protein (MRPS12) in a subset of our samples (n = 28), we found protein levels of MRPS12 tended to correlate with mRNA expression levels (r = 0.29, p = 0.14; Additional file 1: Figure S6).

The declining expression of oxidative phosphorylation genes in monocytes is consistent with previous findings across species [50-53], and from previous transcriptomic studies of aging in human muscle [7,9], and brain [7] tissues. Mitochondrial dysfunctional has been widely reported with aging [54] and many age-related diseases [49]. A major finding of this work is the coordinated down regulation of many oxidative phosphorylation and protein synthesis machinery genes with age in monocytes. However, the potential upstream mechanisms responsible for down-regulation of OXPHOS and protein synthesis genes with age are unclear.

### Potential drivers of an apparent age-related decline in oxidative phosphorylation and protein synthesis machinery gene expression

Six genes with reported regulatory roles for mitochondrial gene expression [55,56] were detectable in our monocyte samples (*PGC-1α, TFB2M, TFAM, MTERF, NRF-2, POLRMT)*, two of which had expression significantly and negatively associated with age (*TFB2M* - transcription factor B2, mitochondrial, FDR = 9.94×10^−9^; and *MTERF -* mitochondrial transcription termination factor, FDR = 1.44×10^−4^). However, there were no detectable transcriptional changes with age for *PCG-1α*, a master regulator of mitochondrial biogenesis, which indirectly up-regulates expression of nuclear OXPHOS genes, mitochondrial protein synthesis machinery, and mitochondrial protein import genes [57].

To identify potential transcriptional regulators for the coordinated expression of oxidative phosphorylation and protein synthesis machinery genes observed in monocytes, we next looked for enrichment [22] of transcription factor binding sites (TFBS) among genes assigned to the ‘blue’ co-expression network module. No TFBS were significantly (FDR < 0.05) enriched among ‘blue’ module genes compared to all monocyte expressed genes. The ‘turquoise’ module on the other hand, which was strongly and negatively correlated with the ‘blue’ module (r < −0.90; Additional file 1: Figure S2), contained a large number of genes (1,466 genes) which were enriched with binding sites for over 50 different transcription factors compared to all monocyte expressed genes (Additional file 2: Table S8). Further, the ‘turquoise’ module included 238 genes with known roles in regulation of transcription (GO:0045449), including a number of transcription factors with expression increasing with older age (*FOXO4, YY1, NFKB1, AHR;* FDR ranging 1.48×10^−7^ – 6.50x10^−4^) and chromatin remodelers which increased with older age (SWI/SNF family genes: *ARID1A, SMARCA4*, *SMARCA2*, *SMARCC2;* FDR ranging 1.10×10^−5^ – 5.94×10^−4^*)* (Additional file 2: Table S9). Future studies may benefit from our identification of several chromatin remodeler and transcriptional modifier genes with expression profiles strongly correlated with an apparent coordinated transcriptional decline of key genes required for mitochondrial biogenesis.

Paradoxically, inhibition of autophagy should reflect an anabolic state and increasing rates of protein synthesis and oxidative phosphorylation [58]; however, in the aging monocyte transcriptome we observed a potential transcriptional decline in autophagy, and a concomitant decline in OXPHOS and protein synthesis gene expression with age. Intriguingly, a decline in AMPK activity with age could potentially explain this paradox, given the dual role of AMPK to activate autophagy and mitochondrial biogenesis [37,57,59]. Similar to the decline in AMPK activity that has been previously reported in aged mice [54], here, we reported decreased expression of the regulatory subunit of AMPK (*PRKAG1*). These results provide clues for further investigations of the role of AMPK dysfunction in aging, and identify potential transcriptional regulators of an age-related decline in oxidative phosphorylation and ribosomal protein synthesis machinery gene expression.

### Epigenomic regulation of age-associated gene expression

To investigate DNA methylation as a potential regulator of the aging transcriptome, we performed lookups using the list of expression-associated methylation sites (eMS) that we recently reported from the same 1,264 monocyte samples [14]. We identified 48% of age-associated genes (1,304 genes, FDR ≤ 0.001) harboring eMS. Methylation profiles were both negatively correlated (69%; range: −0.77, −0.13) and positively correlated (31%; range: 0.13, 0.73) with gene expression profiles. Using mediation analyses to investigate DNA methylation as a potential mediator for the effect of age on gene expression, 17% of age-associated genes (454 genes, FDR ≤ 0.001) were identified harboring CpG sites whose degree of methylation significantly mediated (p_indirect_ < 0.05) the effect of age on gene expression (Additional file 2: Table S10), including a similar percentage of genes from each of the three age-associated pathways: five OXPHOS genes (21%), 18 ribosomal genes (18%), and five autophagy genes (19%) (Table 2). The majority of the mediation effects had similar directions of effect as the overall effect of age on gene expression (85%).

**Table 2:** **Age-associated methylation predicted to mediate the relationship between age and expression of oxidative phosphorylation, ribosome, and autophagy genes**

**Age ~ Methylation**				**Methylation ~** ***cis*** **Gene expression**			**Age ~ Gene expression**					
**CpG ID**	**Chr**	**Cor**	**FDR**	**Gene**	**Cor**	**FDR**	**Indirect**		**Direct**		**Total**	
							**Beta (SE)**	**P-value**	**Beta (SE)**	**P-value**	**Beta (SE)**	**FDR**
***Oxidative phosphorylation genes from gene ontology (GO:0006119):***												
cg07388493	1	−0.4	2.4E-42	*NDUFS5*	0.22	1.1E-12	−0.097 (0.014)	2.7E-12	−0.09 (0.03)	1.9E-03	−0.19 (0.03)	1.2E-08
cg24704287	19	−0.17	1.6E-07	*NDUFB7*	0.16	1.3E-05	−0.037 (0.009)	1.8E-05	−0.11 (0.03)	1.8E-05	−0.15 (0.03)	9.4E-06
cg09267188	8	−0.14	2.1E-05	*UQCRB*	−0.13	1.1E-03	0.028 (0.007)	3.7E-05	0.10 (0.03)	9.3E-05	0.13 (0.03)	1.0E-05
cg27246938	7	−0.11	7.5E-04	*NDUFB2*	−0.24	2.5E-14	0.030 (0.007)	6.1E-05	−0.14 (0.03)	1.1E-07	−0.11 (0.03)	1.2E-04
cg09876992	22	−0.32	3.3E-26	*NDUFA6*	−0.13	8.5E-04	−0.027 (0.007)	1.8E-04	−0.17 (0.03)	7.2E-10	−0.20 (0.03)	6.9E-04
***Ribosome genes from gene ontology (GO:005840):***												
cg17328880	19	−0.25	3.4E-16	*MRPL34*	0.29	4.0E-22	−0.070 (0.01)	7.6E-12	−0.07 (0.03)	4.8E-03	−0.14 (0.03)	3.0E-06
cg04865726	1	−0.27	7.5E-19	*MRPL20*	0.24	1.2E-14	−0.069 (0.011)	7.7E-11	−0.12 (0.03)	3.9E-06	−0.19 (0.03)	9.2E-09
cg08885076	2	−0.2	8.1E-11	*MRPL30*	0.2	7.6E-10	−0.042 (0.008)	6.9E-08	−0.05 (0.03)	5.3E-02	−0.09 (0.03)	1.1E-03
cg16399745	12	−0.29	6.4E-21	*MRPL51*	−0.15	8.4E-05	0.036 (0.008)	5.5E-06	−0.15 (0.03)	1.1E-08	−0.12 (0.03)	6.6E-05
cg16604233	6	−0.26	3.0E-17	*RPS18*	−0.16	4.6E-06	0.042 (0.01)	1.6E-05	−0.16 (0.03)	8.1E-08	−0.11 (0.03)	1.3E-04
cg16000022	11	−0.16	1.8E-06	*MRPL21*	−0.15	4.9E-05	0.028 (0.007)	2.8E-05	0.10 (0.03)	1.3E-04	0.13 (0.03)	6.9E-06
cg17614703	5	0.14	3.2E-05	*CANX*	−0.13	7.7E-04	0.028 (0.007)	2.9E-05	0.10 (0.03)	8.2E-05	0.13 (0.03)	3.2E-05
cg15829665	6	−0.12	5.5E-04	*MRPL18*	−0.13	1.3E-03	0.028 (0.007)	3.6E-05	0.10 (0.03)	9.3E-05	0.13 (0.03)	4.6E-05
cg21252105	9	−0.18	2.8E-08	*MRPL41*	0.15	4.3E-05	−0.035 (0.009)	7.8E-05	−0.10 (0.03)	2.2E-04	−0.13 (0.03)	2.7E-05
cg14663914	19	−0.11	1.1E-03	*RPS15*	0.17	1.7E-06	−0.027 (0.007)	8.9E-05	−0.17 (0.03)	1.5E-09	−0.20 (0.03)	6.5E-06
cg05017994	5	0.16	9.4E-07	*MRPL36*	−0.15	2.3E-05	−0.039 (0.01)	9.6E-05	−0.14 (0.03)	3.2E-07	−0.18 (0.03)	1.9E-07
cg23163653	6	−0.16	3.8E-07	*ABCF1*	−0.13	6.2E-04	−0.027 (0.007)	1.0E-04	−0.17 (0.03)	1.2E-09	−0.20 (0.03)	1.3E-03
cg10700019	8	−0.11	9.8E-04	*RPL8*	−0.28	5.2E-21	0.033 (0.008)	1.1E-04	0.12 (0.03)	7.7E-06	0.15 (0.03)	1.0E-05
cg27209993	6	−0.14	2.7E-05	*MRPS10*	0.17	4.0E-07	−0.023 (0.006)	1.3E-04	−0.06 (0.03)	2.7E-02	−0.09 (0.03)	1.1E-03
cg00435173	17	−0.2	3.7E-10	*RPL27*	0.13	1.1E-03	0.033 (0.009)	1.4E-04	0.12 (0.03)	4.8E-06	0.15 (0.03)	8.0E-04
cg00530414	16	−0.21	8.3E-12	*RPS15A*	−0.15	3.9E-05	0.030 (0.008)	1.8E-04	−0.13 (0.03)	5.0E-06	−0.09 (0.03)	1.0E-03
cg22803868	17	−0.12	6.1E-04	*NUFIP2*	−0.14	2.0E-04	0.023 (0.007)	4.7E-04	0.11 (0.03)	8.8E-05	0.13 (0.03)	7.1E-05
cg13084677	4	0.12	3.8E-04	*RPL9*	0.19	3.5E-09	0.017 (0.006)	4.6E-03	−0.12 (0.03)	1.8E-05	−0.10 (0.03)	7.7E-04
***Autophagy genes from Figure*** 3 ***:***												
cg24213719	18	0.19	1.9E-09	*BCL2*	0.13	7.2E-04	0.028 (0.007)	3.3E-05	0.10 (0.03)	1.4E-04	0.13 (0.03)	1.2E-03
cg11789534	22	−0.17	1.3E-07	*IL2RB*	0.16	4.1E-06	−0.032 (0.008)	4.6E-05	0.09 (0.02)	4.2E-05	0.06 (0.02)	9.9E-04
cg21826784	1	−0.12	3.4E-04	*FRAP1*	−0.13	9.5E-04	−0.027 (0.007)	1.2E-04	−0.17 (0.03)	2.2E-09	−0.20 (0.03)	2.3E-05
cg22117188	3	−0.13	1.4E-04	*PRKCD*	−0.18	1.9E-07	−0.027 (0.007)	1.5E-04	−0.17 (0.03)	5.2E-10	−0.20 (0.03)	8.6E-05
cg18728264	11	−0.19	1.9E-09	*IL10RA*	0.14	3.9E-04	−0.020 (0.006)	4.3E-04	0.21 (0.03)	1.7E-13	0.19 (0.03)	1.5E-08

CpGs whose degree of methylation significantly associated with age (FDR ≤ 0.001), *cis*-gene expression (±1 MB; FDR ≤ 0.001), and was predicted to mediate (indirect p-value < 0.05) the total effect (total beta, SE, FDR) of age on gene expression. Results include only the most significant mediating CpG identified per gene for oxidative phosphorylation genes (from Gene Ontology pathway GO:0006119), ribosome genes (from GO:005840) and autophagy genes (from Figure 3), and are sorted first by pathway, then by the significance of the mediation effect (full mediation results are presented in Additional file 2: Table S10). The direct effects of age on gene expression not supported to be mediated by methylation are also shown (direct beta, SE, p-value). Analyses included 1,264 CD14+ monocyte samples; partial correlations (cor) were adjusted for sex, race, study site, residual contamination with non-targeted cells, and microarray chip effects.

Different from previous studies of the aging transcriptome that did not have measures of DNA methylation, our concurrent transcriptomic and methylomic profiling of the same batch of monocytes allowed us to detect genes harboring CpG sites with methylation profiles which significantly mediated the associations between age and gene expression. These potentially functional CpGs are enriched in predicted enhancer regions compared to all CpGs measured by microarray [14,21], suggesting that DNA methylation of enhancers could play a role in the regulation of gene expression with age. However, we cannot rule out the reverse causality of age-associated expression affecting methylation profiles or uncontrolled (hidden) variation resulting in the correlation between methylation and gene expression. Additionally, the majority of the age and gene expression associations (direct effects) remained significant after adjusting for CpG methylation, suggesting that regulators other than the measured CpG methylation likely contribute to the relationship between age and gene expression. Moreover, further investigations of other potential drivers for gene expression changes with age are warranted.

### Transcriptomic profiles associating with pulse pressure

To see if age-related changes in gene expression may also reflect vascular age, we examined the relationships between age-associated gene expression profiles and a surrogate of vascular age, pulse pressure. Of the 2,704 genes associated with age (FDR ≤ 0.001) in the 1,264 monocyte samples, 15 genes were also associated with pulse pressure (FDR_genome-wide_ ≤ 0.01), after adjusting for age and appropriate biological and technical covariates (Additional file 2: Table S11). The gene most significantly associated with pulse pressure was *PTGER2* (prostaglandin E receptor 2 (subtype EP2)), which had increasing expression associated with higher pulse pressure (FDR_genome-wide_ = 3.15×10^−7^). Additionally, the increasing expression of *MCL1*, a known inhibitor of autophagy [29] and one of the most significant associations we detected with age in monocytes, was also independently associated with higher pulse pressure.

### Transcriptomic profiles associated with age in 423 T cell and monocyte samples

**Figure 5:**
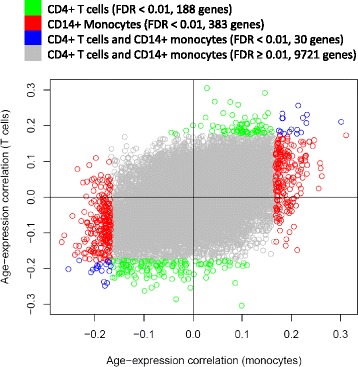
Comparison of the effect of age on gene expression in T cells and monocyte samples. The correlation between age and gene expression is shown in T cells (y-axis), compared to monocytes (x-axis) from 423 individuals, including all 10,322 genes expressed in both T cells and monocytes. Color indicates genes with expression significantly associated with age (FDR < 0.01) in T cells (green, 188 genes) or monocytes (red, 383 genes), both T cells and monocytes (blue, 30 genes), or neither T cells nor monocytes (grey); association analyses were adjusted for race, sex, study site, and residual cell contamination with non-target cells.

We carried out transcriptomic profiling of CD4+ T cell samples using microarrays (Illumina HumanHT-12 v4 Expression BeadChip) for a subset of the MESA samples with transcriptomic data in monocytes (n = 423), and detected 10,322 genes expressed in both T cell and monocyte samples (Figure 1B). A comparison of the effect of age on gene expression in T cells and monocytes is shown in Figure 5, which reveals 188 genes with expression significantly associated with age only in T cells, 383 genes associated with age only in monocyte samples, and 30 genes with age-associated expression in both the T cell and monocyte samples (FDR < 0.01, Additional file 2: Table S12). The majority (93%) of the genes detected with age-associated expression in the subset of 423 monocyte samples were also significantly (FDR ≤ 0.001) associated with age in the full sample of 1,264 monocytes, with similar effect directions observed for all genes (Additional file 1: Figure S7 and Additional file 2: Table S13).

Age-associated genes identified in the subset of 423 monocyte samples were enriched with ribonucleoprotein complex genes, similar to results from the expanded sample size of monocytes. After stratifying by the effect direction, the genes with expression negatively associated with age were enriched with ribonucleoprotein complex and mitochondrion genes, while genes with expression positively associated with age were enriched for cellular biosynthetic processes (Additional file 1: Table S14). The down-regulated mitochondria genes included ATP synthase complex genes (*ATP5E, ATP5S, ATP5G1, ATP5I, ATP5G3*) and electron transport chain genes (*NDUFS5, TXNRD1, NDUFS3, CRYZL1*) which are key genes for oxidative phosphorylation.

No pathways were significantly (FDR < 0.05) enriched among genes with age-associated expression in T cells; however, there was suggestive enrichment for the ribonucleoprotein complex among genes with expression negatively associated with age, and for the immune response pathway among genes with expression positively associated with age (Additional file 1: Table S14). Providing further evidence for a transcriptional decline of ribosomal protein synthesis genes with age in T cells, the majority (62%) of the ribonucleoprotein complex genes with expression profiles negatively associated with age in monocytes were also negatively associated with age in T cells (p < 0.05). However, the overall decline of oxidative phosphorylation gene expression with older age that was detected in monocytes was not detectable in T cells.

These results, from a large number of purified T cell and monocyte samples from the same individuals, identify only a small number of genes with transcriptomic profiles associated with aging in both cell types, supporting the idea that some age-related changes may be cell-type specific. However, the potential decline in protein synthesis machinery gene expression observed in both cell types, and previously reported in human blood leukocytes [10] and brain tissue [6], further support the hypothesis that some transcriptomic changes are conserved to varying degrees across cell types.

### Limitations of the study

Several limitations of the study should be noted. Our investigation included adults aged 55 to 94 years; therefore, these results may not be applicable to younger populations. Also, our primary analysis used microarrays to measure gene expression rather than RNA sequencing, which may have missed low abundance genes. The cross-sectional nature of the investigation also limits inferences for the associations of gene expression with chronological age. Longitudinal analyses are necessary to confirm the effect of age on expression of identified genes and gene networks. We also acknowledge that the analyses of CpG methylation as a potential mediator of the effect of age on gene expression should be interpreted with caution since statistical mediation does not differentiate correlation from causation. Lastly, some of the age-associated transcriptional differences we observed may not reflect differences in protein levels or protein activity, although we have quantified protein levels using western blot for two of our transcriptional signals.

## Conclusions

In this transcriptomic study of purified monocytes from a large, multi-ethnic and mixed gender population, older age appears to be associated with a transcriptomic decline in ribosomal protein synthesis machinery, oxidative phosphorylation, and autophagy pathways. The ability to detect a large number of biologically plausible gene expression changes support the use of CD14+ monocytes, a readily accessible cell population, as a model for further investigations of human aging, including the potential decline of autophagy and mitochondrial biogenesis with age. Our data also provides clues to the potential drivers of these transcriptomic changes with age, such as chromatin remodeler genes and DNA methylation. Further functional work is required to investigate the causes and consequences of these mRNA expression alterations with age.

Our sample size of purified T cells from a subset of the population is also the largest reported to date, which allowed sufficient power to detect age-sensitive genes, and provided suggestive evidence for transcriptomic alterations in ribosomal protein synthesis machinery and immune response pathways with age. The full list of age-genes identified from either CD4+ T cells or CD14+ monocytes harbors many strong candidate genes for future studies of the aging process. In designing such experimental studies one may want to consider that there may be tissue- or cell-specific changes with age, although some patterns of aging are likely similar between different human tissues.

## Methods

### Study population

The Multi-Ethnic Study of Atherosclerosis (MESA) was designed to investigate the prevalence, correlates, and progression of subclinical cardiovascular disease in a population cohort of 6,814 participants. Since its inception in 2000, five clinic visits collected extensive clinical, socio-demographic, lifestyle, behavior, laboratory, nutrition, and medication data [60]. The present analysis is based on analyses of purified monocyte and T cell samples from the April 2010 – February 2012 examination (Exam 5) of 1,264 randomly selected MESA participants (55 – 94 years old, Caucasian (47%), African American (21%) and Hispanic (32%), female (51%)) from four MESA field centers (Baltimore, MD; Forsyth County, NC; New York, NY; and St. Paul, MN). The study protocol was approved by the Institutional Review Boards at Johns Hopkins Medical Institutions, Wake Forest University Health Sciences, Columbia University Medical Center, and the University of Minnesota. All participants signed informed consent.

### Purification of CD14+ Monocytes and CD4+ T cells

Centralized training of technicians, standardized protocols, and extensive quality control (QC) measures were implemented for collection, on-site processing, and shipment of MESA specimens, and routine calibration of equipment was performed. Blood was initially collected in sodium heparin-containing Vacutainer CPTTM cell separation tubes (Becton Dickinson, Rutherford, NJ) to separate peripheral blood mononuclear cells from other elements within two hours from blood draw. Subsequently, monocytes and T cells were isolated with anti-CD14 and anti-CD4 monoclonal antibody coated magnetic beads, respectively, using autoMACS automated magnetic separation unit (Miltenyi Biotec, Bergisch Gladbach, Germany). Initially flow cytometry analysis of 18 specimens was performed, including samples from all four MESA field centers, which were found to be consistently > 90% pure.

### DNA/RNA extraction

DNA and RNA were isolated from samples simultaneously using the AllPrep DNA/RNA Mini Kit (Qiagen, Inc., Hilden, Germany). DNA and RNA QC metrics included optical density (OD) measurements, using a NanoDrop spectrophotometer and evaluation of the integrity of 18 s and 28 s ribosomal RNA using the Agilent 2100 Bioanalyzer with RNA 6000 Nano chips (Agilent Technology, Inc., Santa Clara, CA) following manufacturer’s instructions. RNA with RIN (RNA Integrity) scores > 9.0 was used for global expression microarrays. The median of RIN for our 1,264 samples was 9.9.

### Global expression quantification

The Illumina HumanHT-12 v4 Expression BeadChip and Illumina Bead Array Reader were used to perform the genome-wide expression analysis, following the Illumina expression protocol. The Illumina TotalPrep-96 RNA Amplification Kit (Ambion/Applied Biosystems, Darmstadt, Germany) was used for reverse transcription, and amplification with 500 ng of input total RNA (at 11ul). 700 ng of biotinylated cRNA was hybridized to a BeadChip at 58°C for 16 – 17 hours. To avoid potential biases due to batch, chip, and position effects, a stratified random sampling technique was used to assign individual samples (including five common control samples for the first 480 samples) to specific BeadChips (12 samples/chip) and chip position.

### Epigenome-wide methylation quantification

The Illumina HumanMethylation450 BeadChip and HiScan reader were used to perform the epigenome-wide methylation analysis. The EZ-96 DNA Methylation^™^ Kit (Zymo Research, Orange, CA) was used for bisulfate conversation with 1 μg of input DNA (at 45 μl). 4 μl of bisulfite-converted DNA were used for DNA methylation assays, following the Illumina Infinium HD Methylation protocol. This consisted of a whole genome amplification step followed by enzymatic end-point fragmentation, precipitation, and resuspension. The resuspended samples were hybridized on HumanMethylation 450 BeadChips at 48°C for 16 h. The individual samples were assigned to the BeadChips and to chip position using the same sampling scheme as that for the expression BeadChips.

### Quality control and Pre-processing of microarray data

Data pre-processing and quality control (QC) analyses were performed in *R* (http://www.r-project.org/) using *Bioconductor* (http://www.bioconductor.org/) packages. For expression data, data corrected for local background were obtained from Illumina’s proprietary software GenomeStudio. QC analyses and bead type summarization (average bead signal for each type after outlier removal) were performed using the *beadarray* package [61]. Detection P-values were computed using the negative controls on the array. The *neqc* function of the *limma* [62] package was used to perform a normal-exponential convolution model analysis to estimate non-negative signal, quantile normalization using all probes (gene and control, detected and not detected) and samples, addition of a recommended (small) offset, log_2_ transformation, and elimination of control probe data from the normalized expression matrix. Multidimensional scaling plots showed the five common control samples were highly clustered together and identified three outlier samples, which were excluded subsequently. For both monocyte and T cell assays, we included 2% blind duplicates. Correlations among technical replicates exceeded 0.997.

The Illumina HumanHT-12 v4 Expression BeadChip included >47,000 probes for >30,000 genes (with unique Entrez gene IDs). Statistical analyses excluded probes with non-detectable expression in ≥90% of MESA samples (using a detection p-value cut-off of 0.0001), probes overlapping repetitive elements or regions, probes with low variance across the samples (<10th percentile), or probes targeting putative and/or not well-characterized genes, i.e. gene names starting with KIAA, FLJ, HS, MGC, or LOC.

Bead-level methylation data were summarized in *GenomeStudio*. Because the Illumina HumanMethylation450 BeadChip technology employs a two-channel system and uses both Infinium I and II assays, normalization was performed in several steps using the *lumi* package [63]. We first adjusted for color bias using “smooth quantile normalization”. Next, the data were background adjusted by subtracting the median intensity value of the negative control probes. Lastly, data were normalized across all samples by standard quantile normalization applied to the bead-type intensities and combined across Infinium I and II assays and both colors. QC measures included checks for sex and race/ethnicity mismatches, and outlier identification by multidimensional scaling plots. The final methylation value for each methylation probe was computed as the M-value, essentially the log ratio of the methylated to the unmethylated intensity [64]. The M-value is well suited for high-level analyses and can be transformed into the beta-value, an estimate of the percent methylation of an individual CpG site that ranges from 0 to 1 (thus M is logit(beta-value)).

The Illumina HumanMethylation450 BeadChip included probes for >485,000 CpGs. Statistical analyses excluded CpGs with: “detected” methylation levels in <90% of MESA samples using a detection p-value cut-off of 0.05, existence of any SNPs within 10 base pairs of the targeted CpG, or overlap with a repetitive element or region.

Pre-processing with global normalization removed large position and chip effects across all probes; however, probe-specific chip effects were found for some CpGs and gene transcripts, while probe-specific position effects existed for some CpGs but were ignorable for all gene transcripts. These probe-specific effects were included as covariates in all subsequent analyses.

### Pulse pressure measures

Blood pressure was measured 3 times at 2-minute intervals using an automated oscillometric device (Dinamap Monitor Pro 100, GE Healthcare, Milwaukee, WI) after participants had rested for five minutes in the seated position (MESA Exam 5). Appropriately sized cuffs were used for blood pressure assessment. Blood pressure was defined as the average of the second and third readings. The average systolic and diastolic blood pressure values were used to calculate pulse pressure, which was defined as systolic minus diastolic blood pressure.

### Association analyses

The overall goal of the association analysis was to identify associations, at the genome-wide level, between age and gene expression, age and CpG methylation, and transcript expression and CpG methylation. Association analyses were performed using the linear model (*lm*) function of the *Stats* package and the *stepAIC* function of the *MASS* package in *R*. To identify gene transcripts or methylation sites associated with age, we fit separate linear regression models with age as a predictor of transcript expression or the M-value for each gene transcript or CpG site, respectively. Covariates were sex, race/ethnicity, study site, expression/methylation chip, methylation position (for age-CpG methylation analyses only), and residual sample contamination with non-targeted cells (e.g. non-monocytes, see below). To identify methylation sites associated with gene expression in *cis*, we fit separate linear regression models with the M-value for each CpG site (adjusted for methylation chip and position effects) as a predictor of transcript expression for any autosomal gene within 1 Mb of the CpG in question. Covariates were age, sex, and race/ethnicity, study site, expression chip, and residual sample contamination with non-targeted cells. Sex- and ethnicity-stratified analyses were performed as an internal validation and check of generalizability. To look for potential population stratification, we used EIGENSTRAT [65] to compute principal components (PCs) for each race, based on Affymetrix 6.0 array genotype data [66], and examined the association between the first five PCs and gene expression, as well as CpG methylation, in race stratified analyses. Less than 1% of expression transcripts and CpG methylation sites in monocytes were associated with PCs in the Caucasian and African American populations (FDR < 0.05). However, 14.7% of gene expression transcripts and 3.1% of methylation sites in the Hispanic population were associated with the first two PCs (FDR < 0.05); therefore, analyses in the Hispanic population were adjusted for the first two PCs. P-values were adjusted for multiple testing using the q-value FDR method [67]. The reported FDR was calculated at the genome-wide level for all genes, CpGs, or *cis*-gene/CpGs that were tested.

Association analyses for individual gene transcripts and pulse pressure were performed using the linear model (*lm*) function in R. We fit separate linear regression models with transcript expression as a predictor of pulse pressure. Covariates included age, sex, race/ethnicity, study site, expression/methylation chip, methylation position (for age-CpG methylation analyses only), and residual sample contamination.

To estimate residual sample contamination for monocyte and T cell data analysis, we generated separate enrichment scores for neutrophils, B cells, T cells, monocytes, and natural killer cells. We implemented a Gene Set Enrichment Analysis [68] to calculate the enrichment scores using the gene signature of each blood cell type in the ranked list of expression values for each MESA sample. The cell type-specific signature genes were selected from previously defined lists [69] and passed the following additional filters: at least four-fold more highly expressed in the targeted cell type than in other cell populations and low expression levels in the targeted cells.

### Functional annotation analysis

DAVID Bioinformatics Resources 6.7 was used to examine the enrichment (FDR < 0.05) of GO (Gene Ontology) pathways among gene lists, relative to all genes expressed and passing QC) [22,23]. Experimentally determined protein-protein interactions listed in STRING (Search Tool for the Retrieval of Interacting Genes/Proteins v9.05 and v9.10) [35] were used to create networks of biological connections. Cytoscape [70] was used to visualize protein-protein interactions reported by STRING.

### Weighted gene Co-expression network analyses

For gene network analysis we pre-selected age-associated genes at a less stringent FDR level of 0.01, resulting in a subset of 4,129 genes. To cluster the subset of 4,129 genes into network modules of highly correlated transcripts, we applied the Weighted Gene Co-Expression Network Analysis as implemented in the R package WGCNA [24]. We used this method to first construct a weighted network based on the pairwise correlations among all transcripts considered, using soft thresholding with parameter values chosen to produce approximately a scale-free topology. Then, using the topological overlap measure to estimate the network interconnectedness, the transcripts were hierarchically clustered. We used the default parameters of WGCNA, except for changing the correlation type from Pearson to biweight midcorrelation (which is more robust to outliers) and set the minimum size for module detection from 20 to 10. For each module, we obtained the eigengene (the first eigenvector of the within-module expression correlation matrix, or the first right-singular vector of the standardized within-module expression matrix).

### Stability analysis and consensus modules

Unfortunately, the module structure identified by WGCNA tends to be rather unstable, even when the sample size is relatively large (in the hundreds). Stability of the module structure can be assessed by repeatedly making relatively small, random changes to the data and re-running the analysis, and then assessing the agreement between the resulting structures. One way of making such changes to the data is by sampling random subsets of the data (“sub-sampling”) which contain most but not all of the samples. We performed sub–sampling by obtaining a random sample of 80% of the observations (MESA participants), performing WGCNA on this data subset (with module detection) and repeating this process 200 times. Each of the 200 module assignment was represented by an unsigned network in which all transcripts assigned to the same module were connected by an edge. The Jaccard index [71] was used to evaluate the similarity between any two networks and is equal to the number of edges shared by two networks divided by the total number of edges in present in either network. Hence, the Jaccard index ranges from 0 to 1, with larger values indicating higher similarity between two networks The values of the Jaccard index for the network constructed from the original data and any network obtained from a sub-sampled data set were low with mean value (across 200 replicates) in the range 0.25 - 0.30. To increase the stability of the module assignment, we calculated a consensus network composed of those edges which were present in at least 70% of the 200 networks constructed from the sub-sampled data sets (no minimum size for consensus network modules). We then compared several consensus networks, each based on 200 sub-samples, resulting in Jaccard index values very near 0.90 and indicating much higher stability between consensus networks compared with the (in)stability of networks from individual datasets.

### mRNA quantification using RNA seq

Expression levels accessed by microarray were compared to results from RNA-sequencing in the subset of the monocyte samples (n = 373), indicating excellent reproducibility of microarray data (correlations ranged from 0.45-0.86, median: 0.76). Detailed information describing mRNA quantification is provided in the Additional file 1.

### MCL1 and MRPS12 protein extraction and western blotting

Following DNA/RNA extraction, protein pellet was precipitated from RLT Plus buffer (Qiagen, Inc., Hilden, Germany) with acetone per manufacturer instructions. Pellet was resuspended in 100 μl modified 4× Laemmli buffer [72] (4% SDS, 250 mM Tris HCl, no glycerol, no bromophenol blue, no β-mercaptoethanol) mixed 1:1 with 8 M Urea [73], with SigmaFAST Protease Inhibitor Cocktail Tablet (Sigma-Aldrich, St. Louis, MO). Samples were warmed to 55°C, and sonicated 4 × 30 seconds in a Bioruptor (Diagenode, Denville, NJ). Protein concentration was determined using bicinchoninic acid microplate assay (Thermo Scientific, Rockford, IL). Samples were mixed 1:4 with 5x Loading Buffer Supplement (50% glycerol, 0.02% bromphenol blue, 12.5% β-mercaptoethanol), separated by SDS-PAGE on NuPage Novex 4-12% Bis-Tris Midi gels (Life Technologies, Grand Island, NY), and transferred to Immobilon Fl (Millipore, Billerica, MA) PVDF membranes. Blots were blocked in non-fat dry milk and incubated with antibodies to Mcl1 (Santa Cruz Biotechnology, Santa Cruz, CA) (clone S-19, 1:500 dilution), MRPS12 (Proteintech Group, Chicago, Il) (rabbit polyclonal, catalog #15225-1-AP, 1:333 dilution), and GAPDH (Ablabs, Vancouver, British Columbia) (clone ga1r, 1:3000 dilution) overnight at 4°C. Secondary detection was performed using IRDye 680 and 800 secondary antibodies (LI-COR, Lincoln, NE), and imaged on an Odyssey Classic scanner (LI-COR, Lincoln, NE).

Mcl-1 protein quantification: Mcl-1 often appears as a doublet or triplet in western blot analysis, in agreement with our own observations. These multiple bands are thought to occur for a variety of reasons, including: an alternative initiation site [74], alternative RNA splicing [75], serine/threonine phosphorylation [76,77], and perhaps most notably, and proteolytic cleavage of the N-terminus [74,78,79]. The production, stability, and turnover of Mcl-1 variants is diverse, and thus we chose to focus our quantitation on the dominant, high molecular weight species (40 kDa), which likely corresponds to the full length Mcl-1 protein. GAPDH was used as a loading control because our gene expression analysis showed it has low variance and no association with age (FDR = 0.32). Individual protein band quantification was performed using Image Studio software (LI-COR, Lincoln, NE). Target protein content was corrected for the content of GAPDH in samples.

### Mediation analysis

We performed mediation analysis to investigate the hypothesis that age may have an effect on gene expression mediated through methylation alteration. We used Structural Equation Modeling (SEM) with bootstrapping as implemented in the R package *lavaan* [80] to estimate direct and indirect effects (mediated through DNA methylation) of age on gene expression.

### Availability of supporting data

Microarray data presented in this manuscript has been deposited in the NCBI Gene Expression Omnibus (GEO) repository and is accessible through GEO Series accession number GSE56047. Other supporting data are included in Additional file 1 and Additional file 2.

## Additional files

Additional file 1:
**Figure S1.** Age-associations with the monocyte transcriptome (page 3). **Figure S2.** Correlation between co-expression network modules (page 4). **Figure S3.** Scatterplot of gene expression and age for genes in the ‘black’ co-expression network module (page 5). **Figure S4.** Correlation between *MCL1* expression measured by microarray and RNA-sequencing (page 6). **Figure S5.** MCL1 expression measured using Western Blot (page 7). **Figure S6.** MRPS12 expression measured using Western Blot (page 8). **Figure S7.** Comparison of the effect of age on gene expression in 1,264 monocyte samples compared to results from a subset of 423 samples (page 9). **Table S1.** Population characteristics (page 10). **Table S3.** Gene set enrichment analysis for age-associated genes in monocytes from 1,264 MESA participants (page 11). **Table S4.** Co-expression network modules associated with age (page 12). **Table S14.** Gene set enrichment analysis for age-associated genes in CD4+ T cells and CD14+ monocytes from 423 MESA participants (page 13). Supplementary Methods: **mRNA quantification using RNA seq** (page 14–15). **Supplementary References** (page 16). Additional file 2:
**Table S2.** Association between age and expression of 4,502 age-associated genes (FDR ≤ 0.01) in 1,264 monocyte samples (Tab 1). **Table S5.** Association between age and expression of 54 oxidative phosphorylation genes (Tab 2). **Table S6.** Association between age and expression of 204 ribosomal genes (Tab 3). **Table S7** Association between age and expression of 51 mitochondrial ribosome genes (Tab 4). **Table S8.** Enrichment of transcription factor binding sites (TFBS) among ‘turquoise’ module genes (Tab 5). **Table S9.** Regulators of transcription assigned to the ‘turquoise’ module negatively correlated with ‘blue’ module expression (Tab 6). **Table S10.** Age-associated genes harboring cis-methylation sites associated with age and predicted to mediate the effect of age on cis-gene expression (Tab 7). **Table S11.** Gene expression associated with age (FDR ≤ 0.01) and a pulse pressure (FDR ≤ 0.01) (Tab 8). **Table S12.** Gene expression associated with age (FDR < 0.01) in CD4+ T cells or CD14+ monocytes from 423 individuals (Tab 9). **Table S13.** Comparison of age associations with gene expression in 423 CD14+ monocyte samples and in the expanded CD14+ monocyte sample size (n = 1264) (Tab 10).

## Abbreviations

FDR: False Discovery Rate
CpG: Cytosine-phosphate-guanine dinucleotide
MESA: The Multi-Ethnic Study of Atherosclerosis
WGCNA: Weighted gene co-expression network analysis
Eigengene: The first eigenvector of the within-module expression correlation matrix, or the first right-singular vector of the standardized within-module expression matrix
GO: Gene Ontology
MCL1: Myeloid Cell Leukemia sequence 1
Bcl-2: B-cell CLL/lymphoma 2
TSC22D3: TSC22 domain family, member 3
CEBPD: CCAAT/enhancer binding protein, delta
IL: Interleukin (IL-2, IL-6, IL10RA)
STAT: Signal transducer and activator of transcription
ATG: Autophagy related *(ATG3, ATG5, ATG7)*
MTOR: Mechanistic target of rapamycin
PI3K/Akt: Phosphatidylinositol 3′ -kinase(PI3K)/Akt
JAK: Janus kinase
PDPK1: 3-phosphoinositide dependent protein kinase-1
IL2RB: Interleukin 2 receptor, beta
AMPK: AMP-activated kinase
ATP: Adenosine triphosphate
PRKAG1: Protein kinase, AMP-activated, gamma 1 non-catalytic subunit
BECN1: Beclin-1, autophagy related
ULK1: Unc-51-like kinase 1
HMGB1: High mobility group box 1
CASP: Caspase, apoptosis-related cysteine peptidase (CASP2, CASP8)
FOXO3: Forkhead box O3
OXPHOS: Oxidative phosphorylation
MRP: Mitochondrial ribosome protein (MRPL30, MRPS12)
PGC-1α: Peroxisome proliferator-activated receptor gamma, coactivator 1 alpha
TFB2M: Transcription factor B2, mitochondrial
TFAM: Transcription factor A, mitochondrial
MTERF: Mitochondrial transcription termination factor
NRF-2: GA binding protein transcription factor, alpha subunit 60 kDa
POLRMT: Polymerase (RNA) mitochondrial (DNA directed)
TFBS: Transcription factor binding sites
FOXO4: Forkhead box O4
YY1: YY1 transcription factor
NFKB1: Nuclear factor of kappa light polypeptide gene enhancer in B-cells 1
AHR: Aryl hydrocarbon receptor
SWI/SNF genes: SWItch/Sucrose NonFermentable (ARID1A, SMARCA4, SMARCA2, SMARCC2)
RPS6KA2: Ribosomal protein S6 kinase, 90 kDa, polypeptide 2
NUFIP2: Nuclear fragile X mental retardation protein interacting protein 2
NDUF: NADH dehydrogenase *(NDUFB7, NDUFA6, NDUFAF1)*
MAP1LC3A: Microtubule-associated protein 1 light chain 3 alpha
