# Supplementary material for: Transcriptomic profiles of aging in purified human immune cells
Source: BMC Genomics. 2015 Apr 22;16(1):333. doi: 10.1186/s12864-015-1522-4 (PMC4417516; doi:10.1186/s12864-015-1522-4)
Supplement: Additional file 1: — Figure S1. Age-associations with the monocyte transcriptome (page 3). Figure S2. Correlation between co-expression network modules (page 4). Figure S3. Scatterplot of gene expression and age for genes in the ‘black’ co-expression network module (page 5). Figure S4. Correlation between MCL1 expression measured by microarray and RNA-sequencing (page 6). Figure S5. MCL1 expression measured using Western Blot (page 7). Figure S6. MRPS12 expression measured using Western Blot (page 8). Figure S7. Comparison of the effect of age on gene expression in 1,264 monocyte samples compared to results from a subset of 423 samples (page 9). Table S1. Population characteristics (page 10). Table S3. Gene set enrichment analysis for age-associated genes in monocytes from 1,264 MESA participants (page 11). Table S4. Co-expression network modules associated with age (page 12). Table S14. Gene set enrichment analysis for age-associated genes in CD4+ T cells and CD14+ monocytes from 423 MESA participants (page 13). Supplementary Methods: mRNA quantification using RNA seq (page 14–15). Supplementary References (page 16). [file 12864_2015_1522_MOESM1_ESM.pdf]

ADDITIONAL FILE 1 FOR

Transcriptomic Profiles of Aging in Purified Human Immune Cells

Lindsay M. Reynolds[1], Jingzhong Ding[2], Jackson R. Taylor[3], Kurt Lohman[1], Nicola Soranzo[4], Alberto de la Fuente[5], Tie Fu Liu[2], Craig Johnson[6], R. Graham Barr[7], Thomas C. Register[8], Kathleen M. Donohue[7], Monica V. Talor[9], Daniela Cihakova[9], Charles Gu[10], Jasmin Divers[1], David Siscovick[11], Gregory Burke[1], Wendy Post[9], Steven Shea[7], David R. Jacobs, Jr.[12], Ina Hoeschele[13], Charles E. McCall[2,14], Stephen B. Kritchevsky[2,3], David Herrington[2], Russell P. Tracy[15], Yongmei Liu[1*]

Table of Contents (page 1 – 2)

Supplementary Figures in *Additional File 1*:

Figure S1. Age-associations with the monocyte transcriptome (page 3)

Figure S2. Correlation between co-expression network modules (page 4)

Figure S3. Scatterplot of gene expression and age for genes in the 'black' co-expression network module (page 5)

Figure S4. Correlation between *MCL1* expression measured by microarray and RNA-sequencing (page 6)

Figure S5. MCL1 expression measured using Western Blot (page 7)

Figure S6. MRPS12 expression measured using Western Blot (page 8)

Figure S7. Comparison of the effect of age on gene expression in 1,264 monocyte samples compared to results from a subset of 423 samples (page 9)

Supplementary Tables in *Additional File 1*:

Table S1. Population characteristics (page 10)

Table S3. Gene set enrichment analysis for age-associated genes in monocytes from 1,264 MESA participants (page 11)

Table S4. Co-expression network modules associated with age (page 12)

Table S14. Gene set enrichment analysis for age-associated genes in CD4+ T cells and CD14+ monocytes from 423 MESA participants (page 13)

Supplementary Methods in *Additional File 1*:

mRNA quantification using RNA seq (page 14 – 15)

Supplementary References (page 16)

a

b

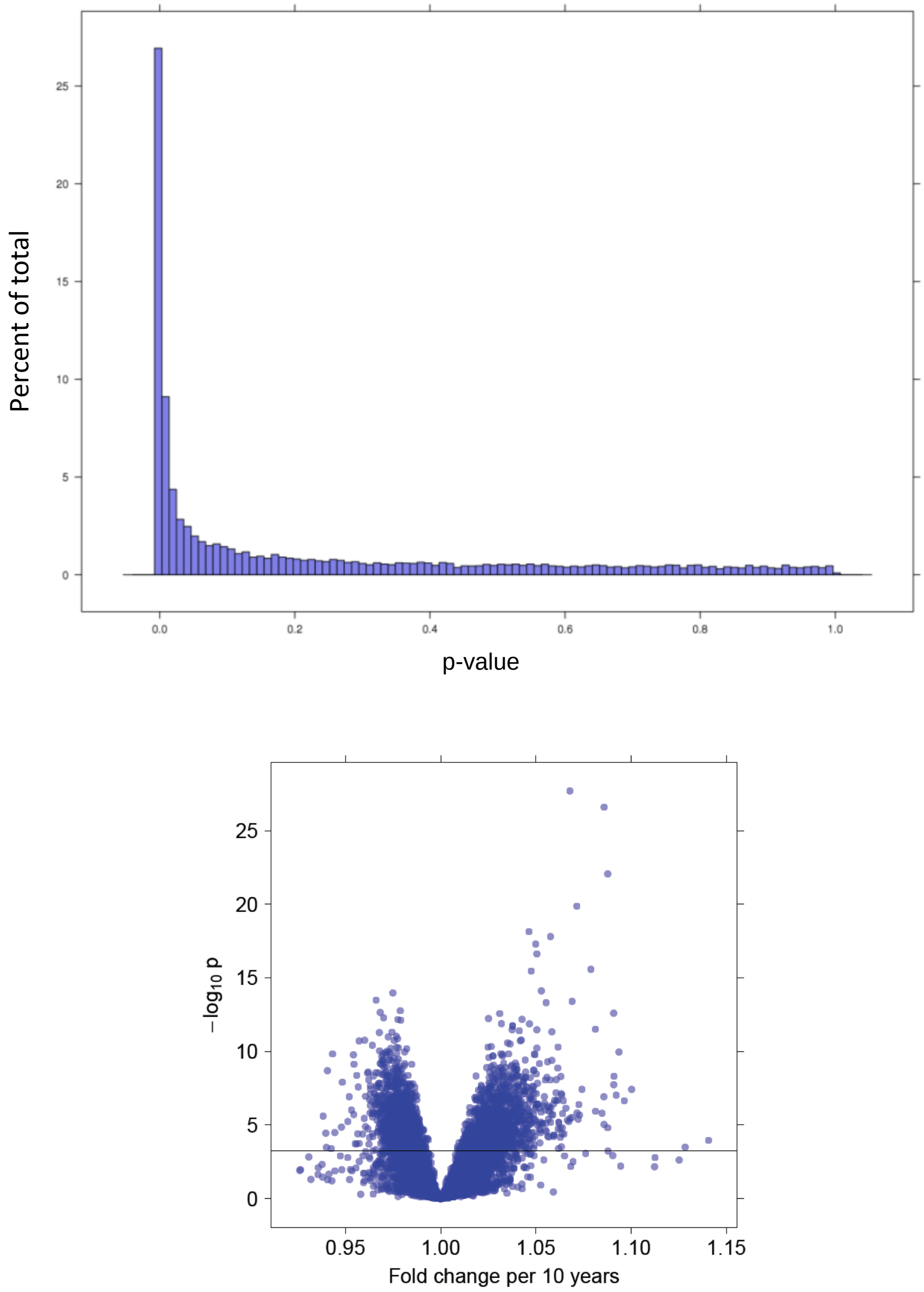

Figure S1. Age-associations with the monocyte transcriptome. A) Histogram of the significance (p-value) of gene expression associations with age in 1,264 CD14+ monocyte samples, including mRNA transcripts from 10,898 genes, measured using the Illumina HumanHT-12 v4 Expression BeadChip14, and **B)** the significance of expression associations with age ($-\log_{10}$ P-values, y-axis) vs. fold change of expression per 10 years (x-axis); the black line represents mRNA expression and age association significance FDR ≤ 0.001; the fold change is derived from linear modeling of gene expression changes ($\log_2$ expression = a + beta X age (per ten year increments); fold change = $2^{beta*10}$

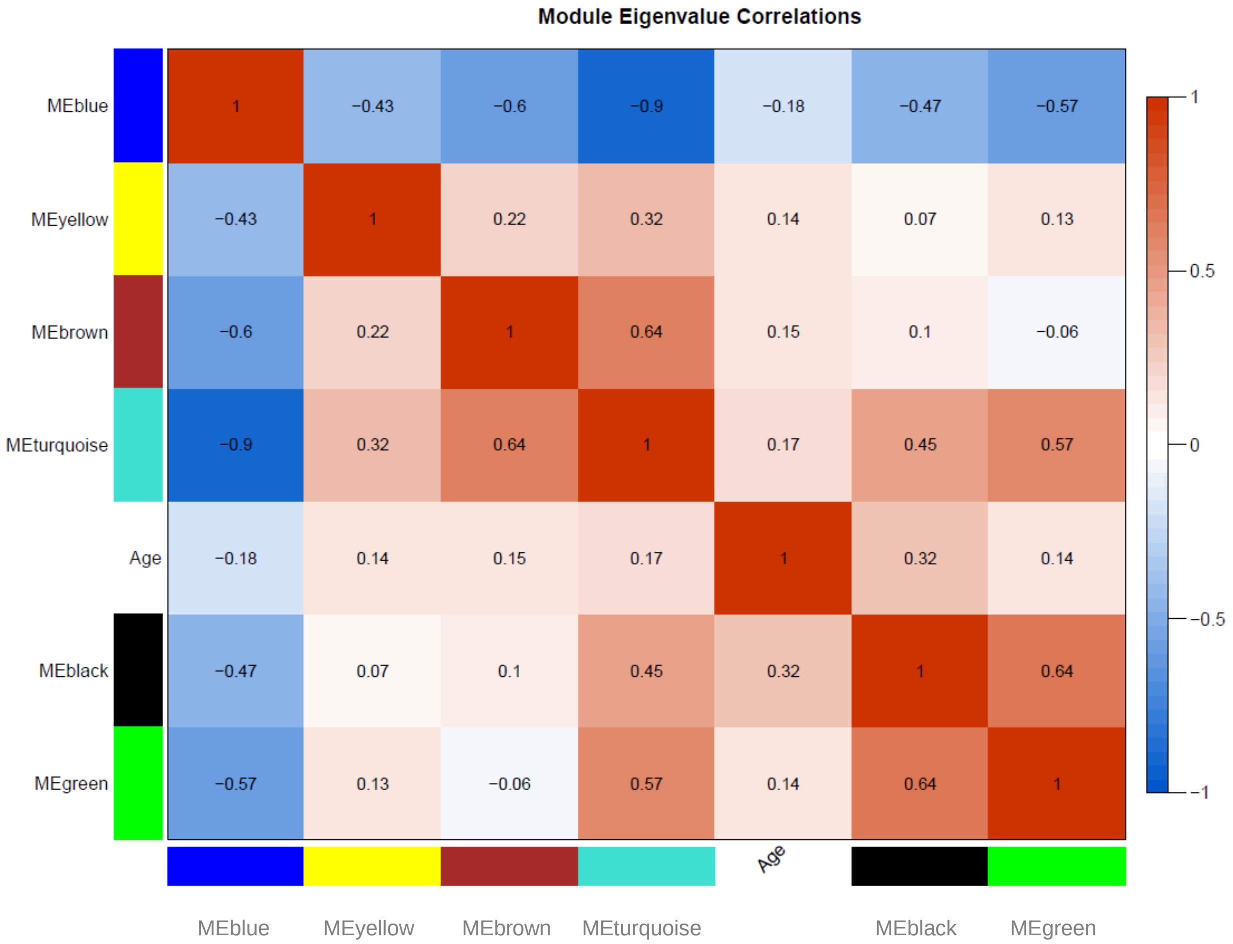

Figure S2. Correlation between co-expression network modules. Pairwise Pearson correlation of six age-associated module eigengenes (MEs) in 1,264 CD14+ monocyte samples; modules detected using a weighted gene co-expression network analysis (WGCNA) including genes with age-associated expression (FDR≤0.01); positive correlations shown in red, negative correlations shown in blue

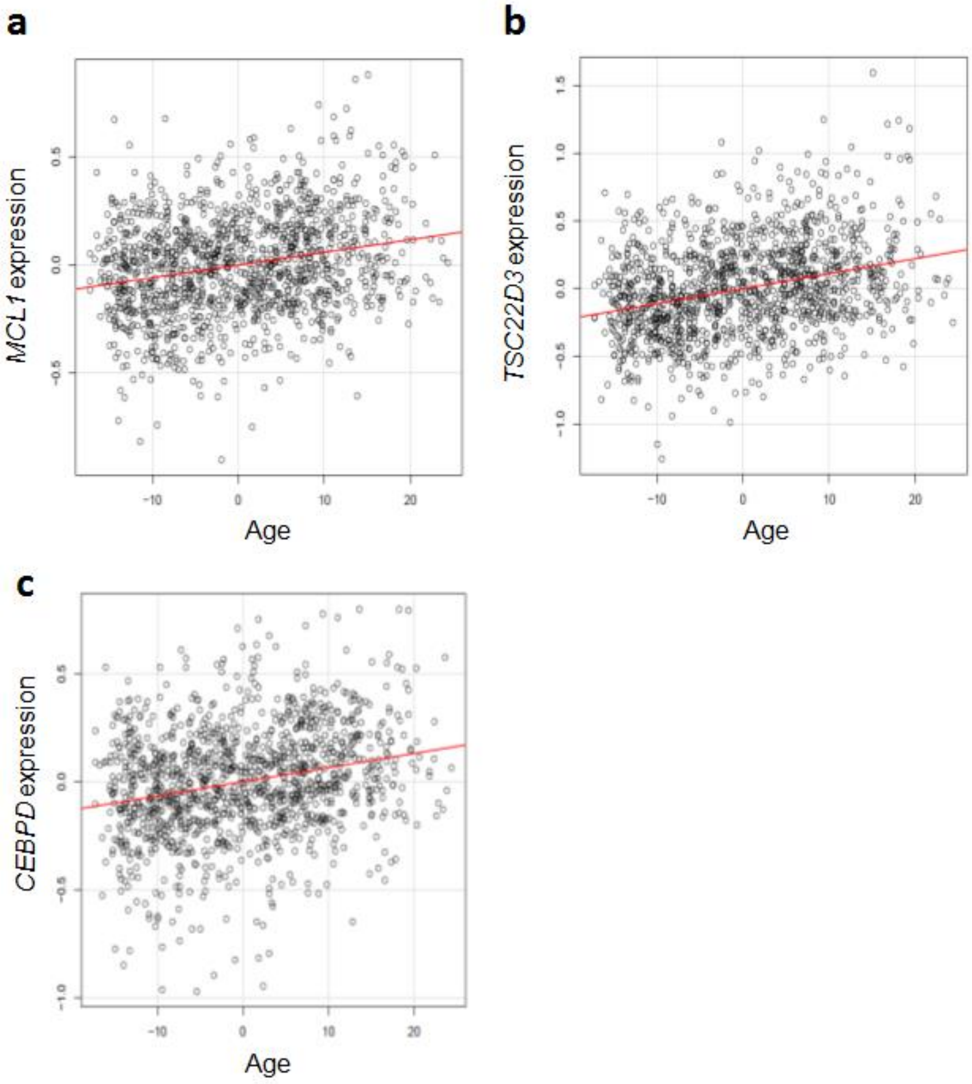

Figure S3. Scatterplot of gene expression and age for genes in the 'black' co-expression network module. Normalized gene expression from 1,264 CD14+ monocyte samples is plotted on the y axis vs. normalized age on the x axis for the 'black' co-expression network module genes: **A)** *MCL1* (myeloid cell leukemia sequence 1; transcript ID: ILMN_1803988; age beta (SE): 0.0065 (0.0007); p: 7.27×10^{-19}; FDR: 7.60×10^{-16}), **B)** *TSC22D3* (TSC22 domain family, member 3; transcript ID: ILMN_2376403; age beta (SE): 0.012 (0.001); p: 2.56×10^{-27}; FDR: 6.69×10^{-24}), and **C)** *CEBPD* (CCAAT/enhancer binding protein, delta; transcript ID: ILMN_1782050; age beta (SE): 0.0070 (0.0008); p: 5.11×10^{-18}; FDR: 3.82×10^{-15})

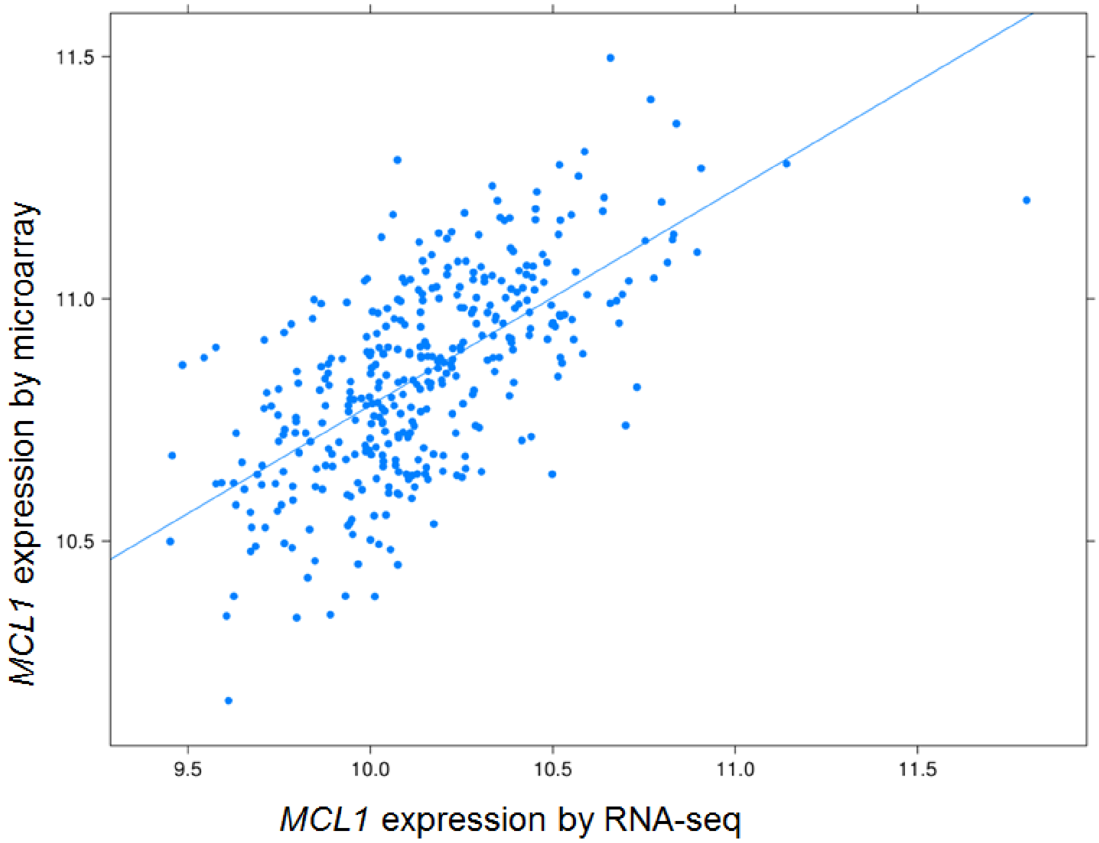

Figure S4. Correlation between *MCL1* expression measured by microarray and RNA-sequencing. Gene expression profiles for *MCL1* (myeloid cell leukemia sequence 1) measured by the Illumina HumanHT-12 v4 Expression microarray (transcript ID: ILMN_1803988; y-axis) are correlated (r = 0.64; p-value = $5.33x10^{-45}$) with *MCL1* expression levels measured by RNA sequencing technology (Ensembl ID: ENSG00000143384; x-axis) in 373 CD14+ monocyte samples.

a

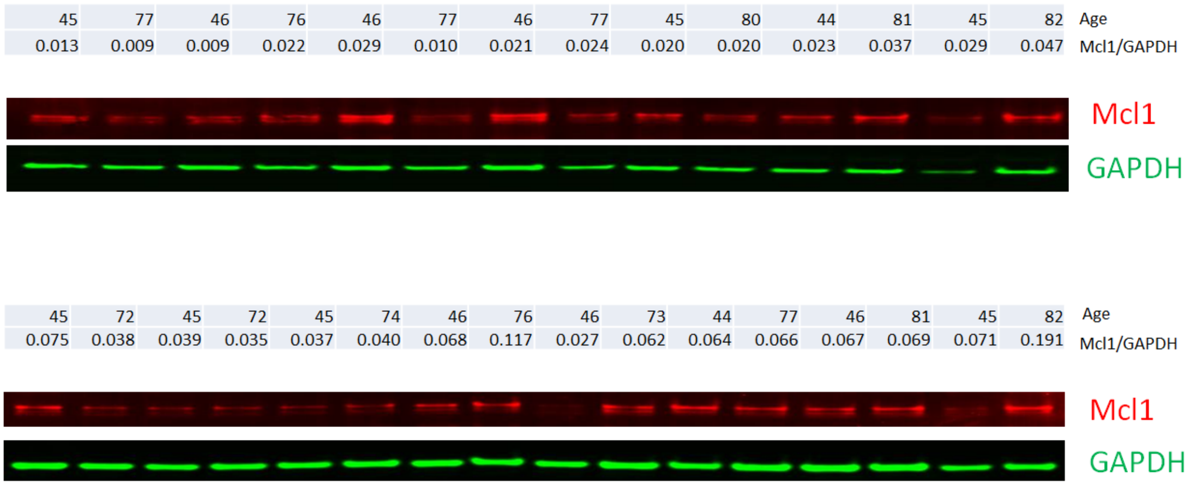

b

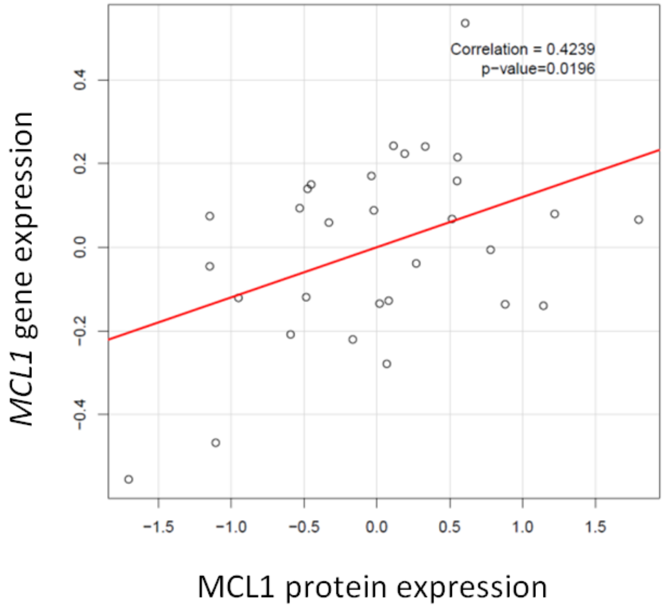

Figure S5. MCL1 expression measured using Western Blot. MCL1 protein expression **a)** measured using Western Blot in 30 MESA CD14+ monocyte samples; **b)** MCL1 protein expression (x-axis) was correlated (0.42, p = 0.02) with *MCL1* gene expression (y-axis, measured by microarray, Illumina ID: ILMN_1803988). Target protein content was corrected for the content of GAPDH in samples.

a

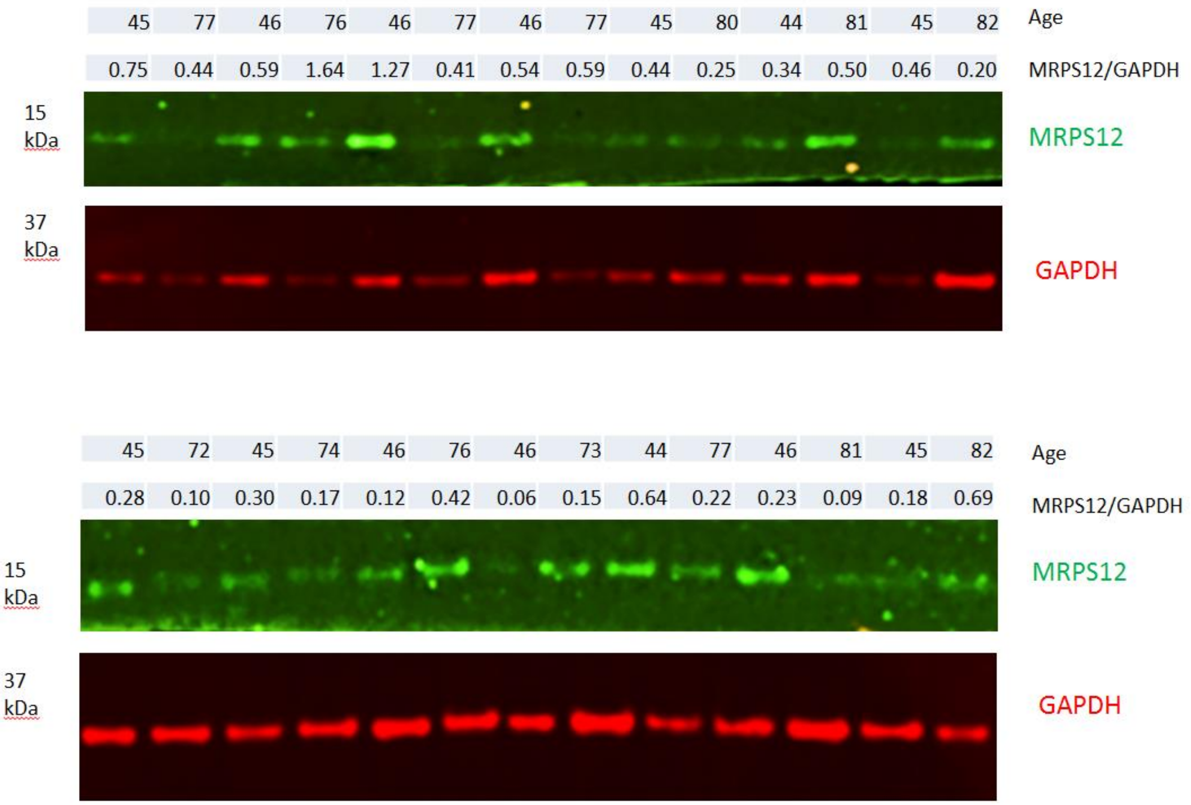

b

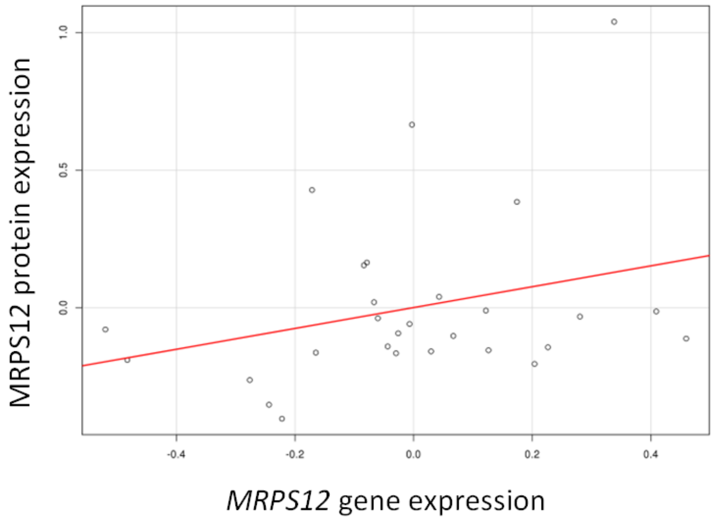

Figure S6. MRPS12 expression measured using Western Blot. MRPS12 (mitochondrial ribosomal protein S12) protein expression **a)** measured using Western Blot in 28 samples; **b)** MRPS12 protein expression (y-axis) tended to be correlated (0.29, p = 0.14) with *MRPS12* gene expression (x-axis; measured by microarray, Illumina ID: ILMN_1714515). Target protein content was corrected for the content of GAPDH in samples.

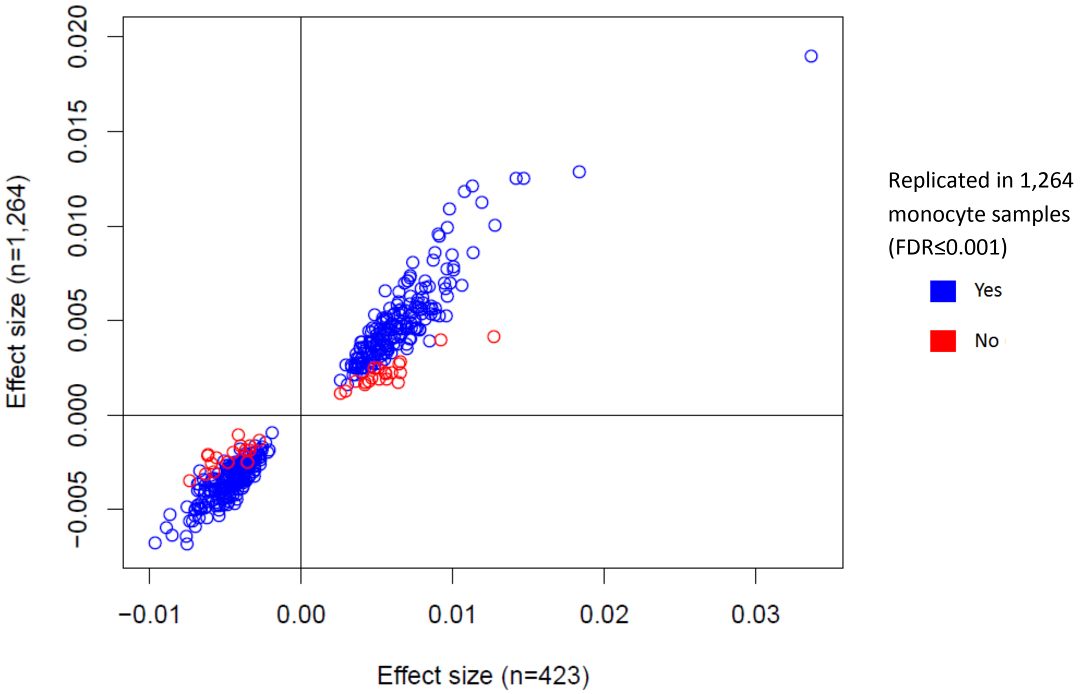

Figure S7. Comparison of the effect of age on gene expression in 1,264 monocyte samples compared to results from a subset of 423 samples. The effect size (beta) associated with age for all 413 genes with expression associated with age (FDR<0.01) in 423 monocyte samples (shown on the x-axis) compared to the effect detected in an expanded sample size of 1,264. Blue circles represent 386 genes that replicated (FDR≤0.001, 93% of genes) in the larger sample size; red circles represent 27 genes that did not replicate (FDR>0.001, 7% of genes) in 1,264 monocyte samples; association analyses adjusted for race, sex, study site, and residual cell contamination with non-target cells

Table S1: Population characteristics

Variable	All (N = 1,264)	Caucasian (N = 590)	Hispanic (N = 402)	African-American (N = 272)
Age (years)	60 ± 10	60 ± 10	59 ± 9	61 ± 9
Women	650 (51%)	285 (48%)	202 (50%)	163 (60%)
Former smoker	358 (50%)	183 (53%)	104 (49%)	71 (44%)
Current smoker	69 (10%)	31 (9%)	18 (8%)	20 (12%)
BMI (kg/m^2)	30 ± 6	29 ± 5	30 ± 5	31 ± 6
Pulse pressure (mmHg)	58 ± 18	56 ± 17	57 ± 18	62 ± 18
Hypertension	596 (61%)	278 (55%)	163 (58%)	155 (78%)
Diabetic	289 (23%)	81 (14%)	106 (26%)	102 (38%)

Mean ± standard deviation provided for continuous variables; count (percentage) provided for discrete variables for all participants included in analysis, and by race

Table S3. Gene set enrichment analysis for age-associated genes in monocytes from 1,264 MESA participants

a) Down-regulated

Gene ontology pathway	Gene Ontology ID	Gene count	Fold enrichment	Nominal P-value	FDR
Structural constituent of ribosome	GO:0003735	79	4.8	3.88E-36	6.00E-33
Mitochondrion	GO:0005739	228	2.1	3.90E-34	5.57E-31
Ribosome	GO:0005840	94	3.7	1.83E-33	2.61E-30
Ribonucleoprotein complex	GO:0030529	150	2.6	7.96E-31	1.14E-27
Translation	GO:0006412	97	2.8	7.42E-23	1.30E-19
Mitochondrial ribosome	GO:0005761	35	5.6	2.67E-20	3.81E-17
RNA processing	GO:0006396	102	1.9	1.31E-10	2.31E-07
Oxidative phosphorylation	GO:0006119	29	3.4	5.04E-09	8.85E-06

b) Up-regulated

Gene ontology pathway	Gene Ontology ID	Gene count	Fold enrichment	Nominal P-value	FDR
Transcription regulator activity	GO:0030528	188	1.5	1.08E-10	1.69E-07
Protein amino acid phosphorylation	GO:0006468	96	1.8	1.42E-08	2.57E-05
Cytoskeleton	GO:0005856	137	1.6	3.50E-08	5.06E-05
Intracellular signaling cascade	GO:0007242	152	1.5	5.39E-08	9.75E-05
Regulation of small GTPase mediated signal transduction (Ras/Rho)	GO:0051056	46	2.2	1.44E-07	2.60E-04
GTPase regulator activity	GO:0030695	64	1.8	1.90E-06	2.97E-03
Nuclear lumen	GO:0031981	188	1.3	9.55E-06	1.38E-02
Response to insulin stimulus	GO:0032868	22	2.8	1.29E-05	2.32E-02

Enrichment analysis included 1,330 genes with expression negatively associated with age (down-regulated; FDR≤0.001), and 1,374 genes with expression positively associated with age (up-regulated; FDR≤0.001); relative to a background of 10,898 genes with expression detected in 1,264 CD14+ monocyte samples

Table S4. Co-expression network modules of age-associated genes associated with chronological age

Co-expression network modules		Age		
Module (gene count)	Pairwise correlation: absolute median [range]	Cor	Percent variance	P-value
Black (3)	0.62 [0.45, 0.90]	0.31	9.7	1.8E-30
Blue (217)	0.42 [-0.69, 0.93]	-0.18	3.1	2.1E-10
Turquoise (1,466)	0.44 [-0.80,0.96]	0.17	2.7	2.6E-09
Brown (42)	0.42 [-0.60,0.89]	0.14	2.0	4.1E-07
Yellow (42)	0.41 [-0.54,0.94]	0.14	1.9	6.9E-07
Green (42)	0.45 [-0.65,0.95]	0.14	1.8	1.3E-06

Mutually exclusive gene modules with coordinate expression profiles associated with chronological age were identified using weighted gene co-expression network analysis (WGCNA), including all genes with age-associated expression (FDR $\leq$ 0.01) in 1,264 CD14+ monocyte samples. For each module, the number of genes assigned to that module is reported, along with the absolute median pairwise correlation (and range) between genes within each module. The partial correlation (cor), percent variance, and significance (P-value) are reported for each module from the association of the module eigengene and age; covariates included: race, sex, site of data collection, and residual sample contamination with non-targeted cells (see **Methods**)

Table S14. Gene set enrichment analysis for age-associated genes in CD4+ T cells and CD14+ monocytes from 423 MESA participants

a) Down-regulated

Enriched pathway	Term ID	Gene count	Fold enrichment	Nominal P-value	FDR	Genes
**CD4+ T cells**						
ribonucleoprotein	SP_PIR_KEYWORDS	11	3.1	2.99E-03	3.6	MRPL10, MRPS34, DKC1, RPL3, MRPL47, MRPL39, SRPRB, RPS4X, SNRPF, NHP2, FBL
**CD14+ Monocytes**						
ribonucleoprotein complex	GO:0030529	30	3.0	1.07E-07	1.34E-04	MRPS35, RPL14, NHP2L1, MRPS12, PPIL1, MRPS11, IMP3, UTP11L, MRPL17, MRPL36, WDR12, TAF9, MRPL39, IMP4, MRPL33, APEX1, MRPL35, MRPL51, MRPS23, EMG1, SRPRB, SLBP, PPIH, EIF2S1, RPS13, LSM10, MRPL45, SIP1, MRPL46, NHP2
mitochondrion	GO:0005739	39	2.2	2.58E-06	3.26E-03	MRPS35, ATP5E, MRPS12, PNKD, MRPS11, UROS, RG9MTD1, STOML2, MPV17, PMAIP1, ATP5G1, CCDC56, ATP5G3, SDHAF1, TRIAP1, NDUFS5, MRPL17, ATP5S, MCEE, SLC25A46, MRPL36, TFB2M, MRPL39, NDUFS3, ATP5I, MRPL33, MRPL35, ABCE1, MRPL51, MRPS23, C14ORF156, AK2, TMEM126A, TST, COQ3, ISCA2, SLC25A19, MRPL45, MRPL46

b) Up-regulated

Enriched pathway	Term ID	Gene count	Fold enrichment	Nominal P-value	FDR	Genes
**CD4+ T cells**						
immune response	GO:0006955	12	3.7	2.90E-04	0.45	LILRB2, CYBB, CD86, KYNU, C5AR1, RGS1, AQP9, LYN, LTB4R, TAP2, CXCL16, LILRA5
**CD14+ Monocytes**						
positive regulation of cellular biosynthetic process	GO:0031328	21	2.9	2.81E-05	0.045	KLF6, IRS2, FOXO1, CREB5, AFF1, FOXO3, FOXO4, AHR, STAT3, NRIP1, CITED2, CHD8, MTF1, ETS2, HIPK2, SMARCD1, USP21, MKL1, THBS1, AKIRIN2, SERTAD2

Results from enrichment analysis of age-associated expression in T cells and monocytes (using DAVID, FDR<0.05); analysis in T cells included 137 genes with expression negatively associated with age (down-regulated, (FDR<0.01), and 81 with expression positively associated with age (up-regulated, FDR<0.01); analysis in monocytes included 221 down-regulated (FDR<0.01), and 192 up-regulated genes (FDR<0.01); background gene list included all 10,322 genes detected in both CD4+ T cells and CD14+ monocytes from the same 423 MESA participants.

Supplementary Methods in *Additional File 1*:

mRNA quantification using RNA seq

Total RNA samples were enriched for mRNA, by depleting rRNA using the MICROBExpress kit from Ambion and following the manufacturer's instructions. Poly(A) mRNA was enriched, and Illumina compatible, strand-specific libraries were constructed using Illumina's TruSeq Stranded mRNA HT Sample Prep Kit (Illumina, RS-122-2103). 1 ug of total RNA with RIN ≥ 8.0 was converted into a library of stranded template molecules suitable for subsequent cluster generation and sequencing by Illumina HiSeq. The libraries generated were validated using Agilent 2100 Bioanalyzer and quantitated using Quant-iT dsDNA HS Kit (Invitrogen) and qPCR. Six individually indexed cDNA libraries were pooled and sequenced on Illumina HiSeq, resulting in an average of close to 30 million reads per sample. Libraries were clustered onto flow cells using Illumina's TruSeq PE Cluster Kit v3 (PE-401-3001) and sequenced 2X100 cycles using TruSeq SBS Kit -HS (FC-401-3001) on an Illumina HiSeqTM 2500. A total of 64 lanes were run to generate approximately 30 million 2 x 101 Paired End reads per sample. The Illumina HiSeq Control Software (HCS v2.0.12) with Real Time Analysis (RTA v1.3.61) was used to provide the management and execution of the HiSeq 2500. Illumina sequencing runs were processed to de-multiplex samples and generate FastQ files using the Illumina provided *configureBclToFastq.pl* script to automate running CASAVA 1.8.4 using default parameters for removal of sequencing reads failing the chastity filter (yes) and mismatches in the barcode read (0). Following generation of FastQ files, reads were trimmed to remove poor quality reads (or read tails) using *Btrim* (5 base sliding window average with $Q >$ 15) [1] and then trimmed to remove any adaptor sequence present in the reads using custom perl scripts (trim sequences containing 11 base tag of adaptor, final length >40 bases). The *Ensembl* GRCh37 *Homo Sapiens* reference file, annotations and Bowtie2 indexes were downloaded from the *igenomes.com* website (10-Apr-2013) for mapping of the sequencing reads to the genome and read counting. *Bowtie2 (2.1.0)* and *TopHat2 (2.0.8)* were used to map the sequencing reads to the genome using a mate-inner-distance of 100 bp and '*firststrand*' options [2, 3] . Following alignment, *bam* files were merged using the *samtools* (0.1.19) merge function [4], and read counts per gene were obtained using *HTSeq* (0.5.4p3) (http://www-huber.embl.de/users/anders/HTSeq /doc/ overview.html). The '*intersection-strict*' overlap resolution mode and '*stranded reverse*' options were used in *HTSeq*.

Data pre-processing and QC analyses were performed in *R* (http://www.r-project.org/) using *Bioconductor* (http://www.bioconductor.org/) packages. The transcript-based raw count data files for each sample from *TopHat2* were combined into a count matrix with 56,303 features (rows) and 374 MESA samples (columns). The median total count per sample was 28.8 million. Reads denoted by *TopHat2* as "no_feature","ambiguous", "too_low_aQual", "not_aligned", "alignment_not_unique" were removed. Counts were converted to Counts Per Million (CPM) using the *cpm* function of the *edgeR* package [5] , and all features with CPM ≤ 0.25 in ≥90% of the 374 MESA samples were removed. Features assigned to the mitochondrial genome were removed as well. Using the *biomaRt* package and querying the *Ensembl BioMart* database, *Entrez Gene ID*s, Gene Symbols, genome coordinates, gene length and percent GC content were obtained for 12,585 features which had a corresponding *Entrez ID or* Illumina HumanHT-12 v4 probe ID. To be able to continue to use the flexible and computationally efficient linear modeling functions in *R,* we transformed the raw count data to log counts per million (y = logCPM) as recommended by Law et al (2013) [6]:

$$y_{gs} = log_2\left(\frac{c_{gs} + 0.5}{T_s + 1} \cdot 10^6\right)$$

where c_{gs} is the raw count of gene transcript g in sample s, and T_s is the normalized total count of sample s, using the Trimmed Mean of M-values (TMM) normalization method [7] as implemented in the *calcNormFactors* function in the *edgeR Bioconductor* package [5]. We either performed only this TMM normalization, or we applied quantile normalization (QN) to the logCPM values. Because the logCPM values' variance tends to decrease with increasing count for smaller counts, we used the voom function of the *limma* package [8] to estimate the mean-variance trend non-parametrically and to predict the residual variance of each individual observation for each gene. Then we incorporated the inverse residual variances into the linear modeling (*lm*) as weights in a standard manner. For the logCPM data, we imposed the same low variance filter that we had used for the microarray data, removing another 192 features with the lowest variance and retaining 12,380 features for analysis. We then performed weighted linear model analyses with the otherwise exact same models as for the microarray data.

Supplementary References in *Additional File 1*

1. Kong Y: **Btrim: a fast, lightweight adapter and quality trimming program for next-generation sequencing technologies**. *Genomics* 2011, **98:**152-153.

2. Langmead B, Trapnell C, Pop M, Salzberg SL: **Ultrafast and memory-efficient alignment of short DNA sequences to the human genome**. *Genome Biol* 2009, **10:**R25.

3. Trapnell C, Pachter L, Salzberg SL: **TopHat: discovering splice junctions with RNA-Seq**. *Bioinformatics* 2009, **25:**1105-1111.

4. Li H, Handsaker B, Wysoker A, Fennell T, Ruan J, Homer N, Marth G, Abecasis G, Durbin R: **The Sequence Alignment/Map format and SAMtools**. *Bioinformatics* 2009, **25:**2078-2079.

5. Robinson MD, McCarthy DJ, Smyth GK: **edgeR: a Bioconductor package for differential expression analysis of digital gene expression data**. *Bioinformatics* 2010, **26:**139-140.

6. Law CW, Chen Y, Shi W, Smyth GK: **Voom! Precision weights unlock linear model analysis tools for RNA-seq read counts.**; 2013.

7. Robinson MD, Oshlack A: **A scaling normalization method for differential expression analysis of RNA-seq data**. *Genome Biol* 2010, **11:**R25.

8. Smyth GK: **Limma: linear models for microarray data.** In *Bioinformatics and Computational Biology Solutions using R and Bioconductor*. Edited by Gentleman R, Carey V, Dudoit S, Irizarry R, Huber W. New York: Springer; 2005:397-420.
